# Global, regional, and national epidemiology of childhood neuroblastoma (1990–2021): a statistical analysis of incidence, mortality, and DALYs

**DOI:** 10.1016/j.eclinm.2024.102964

**Published:** 2024-12-06

**Authors:** Jusen Nong, Cheng Su, Changhua Li, Congjun Wang, Wei Li, Yong Li, Peng Chen, Yanqiang Li, Zihao Li, Xinjin She, Zuxin Yuan, Sentian Liu, Chao Chen, Qian Liao, Yige Luo, Bo Shi

**Affiliations:** a Department of Pediatric Surgery, The First Affiliated Hospital of Guangxi Medical University, Nanning, 530021, Guangxi Zhuang Autonomous Region, PR China; b Department of Emergency, The First Affiliated Hospital of Guangxi Medical University, Nanning, 530021, Guangxi Zhuang Autonomous Region, PR China; c Department of Thoracic Surgery, Liuzhou People's Hospital Affiliated to Guangxi Medical University, Liuzhou, Guangxi Zhuang Autonomous Region, PR China; d Department of Epidemiology and Biostatistics, School of Public Health, Guangxi Medical University, Nanning, 530021, Guangxi Zhuang Autonomous Region, PR China

**Keywords:** Neuroblastoma, Global burden of disease study, Incidence, Mortality, Childhood

## Abstract

**Background:**

Neuroblastoma is the most prevalent extracranial solid tumor in pediatric populations worldwide, representing 8–10% of childhood malignancies and contributing to approximately 15% of pediatric cancer-related fatalities. This study aims to report global trends in the incidence, mortality, and disability-adjusted life years (DALYs) of childhood neuroblastoma from 1990 to 2021.

**Methods:**

The study utilized data from the Global Burden of Disease (GBD) database to analyze neuroblastoma incidence, mortality, and DALYs in children aged 0–14 years. Rates for incidence, mortality, and DALYs were calculated per 100,000 population, with 95% uncertainty intervals (UIs). Data from 204 countries and territories were stratified by age, sex, and location. Trends were assessed using Joinpoint regression models to compute the annual percent change (APC) and log-transformed linear regression models to calculate the estimated average annual percentage change (EAPC).

**Findings:**

Globally, the incidence of neuroblastoma in children in 2021 was 5560 cases (95% UI, 3734.21–7560.03), with 1977 deaths (95% UI, 1445.04–2528.54), and 174,186.30 DALYs (95% UI, 127,104.64–223,265.92). From 1990 to 2021, the incidence increased by 30.26% (95% UI, −1.24% to 72.51%), mortality by 20.35% (95% UI, −12.44% to 63.30%), and DALYs by 20.08% (95% UI, −12.89% to 63.27%). The incidence rate rose from 0.25 (95% UI, 0.18–0.33) per 100,000 individuals in 1990 to 0.28 (95% UI, 0.19–0.38) per 100,000 individuals in 2021, an overall increase of 12.60% (95% UI, −14.62% to 49.12%). Among the five Sociodemographic Index (SDI) regions, the highest EAPCs were observed in the low-to-mid SDI regions for incidence (1.87%; 95% CI, 1.64%–2.10%), mortality (1.22%; 95% CI, 1.09%–1.34%), and DALYs (1.36%; 95% CI, 1.15%–1.57%). Regionally, Central Asia exhibited the fastest annual increase in incidence (EAPC = 2.76%; 95% CI, 2.18%–3.34%). At the national level, India had the highest number of neuroblastoma cases globally in 2021, with 685 cases (95% UI, 404.16–1007.67).

**Interpretation:**

The global trends for incidence, mortality, and DALYs related to pediatric neuroblastoma initially increased and then decreased, although an overall increasing trend was observed. However, the burden of disease remains significant in low-, low-middle-, and middle-SDI regions. A comprehensive understanding of the epidemiology of neuroblastoma in children is crucial for enhancing disease prevention and control efforts.

**Funding:**

This research was funded by the Guangxi Natural Science Foundation (Grant No. 2024GXNSFAA010420) and the 10.13039/501100012543Youth Science Foundation of Guangxi Medical University (Grant No. GXMUYSF202404).

**Research in context**

**Evidence before this study**

We conducted a comprehensive literature search using the specific terms “neuroblastoma,” “children,” “epidemiology,” and “Global Burden of Disease (GBD)” in both PubMed and Web of Science, up to September 2024. To date, epidemiological studies on childhood neuroblastoma have primarily originated from regional or national datasets, as well as from the International Agency for Research on Cancer (IARC). In 1992, IARC published a global epidemiological report on neuroblastoma, which included data from over 50 countries. This report emphasized that neuroblastoma incidence rates were higher in regions with greater economic development, and racial disparities were identified as significant contributors to differences in incidence.

**Added value of this study**

As part of the GBD 2021 study, we have, for the first time, systematically analyzed large-scale data on the incidence, mortality, and disability-adjusted life years (DALYs) for childhood neuroblastoma across 204 countries and regions from 1990 to 2021. Our study reveals that global rates of incidence, mortality, and DALYs associated with childhood neuroblastoma initially increased from 1990 to 2019 but have shown a decline since 2019, despite an overall upward trend. Differences were observed based on socioeconomic development levels, with a continuous reduction in disease burden in high and upper-middle sociodemographic index (SDI) regions, while low, lower-middle, and middle SDI regions experienced an increase in burden.

**Implications of all the available evidence**

The findings of this study provide updated and reliable evidence on the global burden of neuroblastoma, offering valuable insights for healthcare professionals worldwide, particularly pediatricians, oncological researchers, and public health policymakers. Physicians and researchers must continue to improve screening, diagnosis, treatment, and management strategies for neuroblastoma, fostering the development of innovative therapies. Public health policymakers can leverage these data to formulate targeted prevention and control strategies to alleviate the burden of this disease in the future.

## Introduction

Neuroblastoma (NB) is the most prevalent extracranial solid tumor in children globally, accounting for 8–10% of pediatric malignancies and contributing to 15% of childhood cancer fatalities.^1^, ^2^, ^3^ This embryonal tumor originates from the sympathetic nervous system, frequently manifesting in the adrenal medulla and paravertebral ganglia, leading to lesions in the neck, chest, abdomen, or pelvis.^4^^,^^5^ The clinical manifestations of neuroblastoma are highly variable, ranging from asymptomatic masses to life-threatening disseminated disease, and in some cases, spontaneous regression occurs. As one of the most common malignant tumors in early childhood, neuroblastoma is typically diagnosed at a median age of 17–18 months, with incidence peaking during the first year of life and declining with age.^6^ Approximately 90% of cases are diagnosed in patients under five years of age, with a notable concentration of cases in children younger than fourteen years old. Epidemiological studies have indicated that neuroblastoma incidence is higher in regions with elevated standards of living and certain ethnic groups, while it is relatively lower in much of South and East Asia, including China, Japan, and India.^7^ In the United States, the prevalence is 8.5 per million among black children compared to 11.5 per million among white children.^7^ To date, there has not been a comprehensive global study reporting on the incidence and mortality trends of neuroblastoma. The latest Global Burden of Disease (GBD) 2021 report introduces new data on the global burden of neuroblastoma and other peripheral nerve tumors.^8^ This study utilizes the GBD database to analyze and statistically report the incidence, mortality, and disability-adjusted life years (DALYs) of neuroblastoma and other peripheral nerve tumors in children aged 0–14 years from 1990 to 2021. Given that neuroblastoma predominantly affects children and peripheral nerve tumors are rare in this age group, the results primarily reflect the global disease burden of neuroblastoma in children. The findings of this study aim to provide data to support the development of new diagnostic and therapeutic strategies worldwide, with the ultimate goal of reducing the global burden of this disease.

## Methods

### Overview and methodological details

The GBD data source is recognized as one of the most comprehensive and systematic global epidemiological efforts. It is led by the Institute for Health Metrics and Evaluation (IHME) at the University of Washington, with the objective of quantifying health losses caused by various diseases, injuries, and risk factors.^9^ The GBD framework enables comparative assessments of incidence and mortality across different countries, regions, and globally. In GBD analyses, three key indicators are employed to quantify disease burden: mortality, incidence, and DALYs. DALYs are calculated as the sum of Years of Life Lost (YLL) due to premature death and Years Lived with Disability (YLD). The specific formulas used are:

(1) YLL=Numberofdeaths×Standardlifeexpectancyattheageofdeath

(2) YLD=Prevalenceofthecondition×Disabilityweight

Disability weights are assigned based on expert consensus, with values ranging from 0 (indicating perfect health) to 1 (indicating death). This comprehensive methodology allows for a scientifically grounded understanding of the global burden of diseases and injuries. This study retrieved and analyzed data on neuroblastoma cases, incidence, mortality, and DALYs in children aged 0–14 years from 1990 to 2021. The data were sourced from the GBD database (https://vizhub.healthdata.org/gbd-results/), encompassing 204 countries and territories.^8^ The GBD data utilized in our analysis were downloaded on 3 July 2024. The analysis included multiple dimensions such as sex, age (categorized as under 1 year, 1–2 years, 2–4 years, 5–9 years, and 10–14 years), and location. However, race and ethnicity-related analyses were not conducted due to the unavailability of such parameters in the GBD database. This cross-sectional study, which involves the analysis and description of disease data over time and across regions, does not include identifiable personal information. Consequently, the Ethics Committee of Guangxi Medical University approved a waiver for the informed consent procedure. The study was conducted in strict adherence to the Strengthening the Reporting of Observational Studies in Epidemiology (STROBE) guidelines.^10^

### Sociodemographic index

Sociodemographic Index (SDI) serves as a comprehensive measure of a country's or region's socio-economic development.^11^ This index encompasses various dimensions, including but not limited to, the economic structure and size, educational attainment, living standards, and social welfare and security. The index values range from 0 to 1, with higher values indicating greater socio-economic development. According to the GBD database, countries and regions are categorized into five classes based on their SDI: low, low-middle, middle, middle–high, and high. This classification facilitates the study of the impact of socio-economic indices and geographic disparities on the burden of neuroblastoma in children.

### Statistical analysis

Incidence, mortality, and DALYs rates, along with their 95% uncertainty intervals, were calculated per 100,000 population according to the GBD database statistics. Annual percent change (APC) and its 95% confidence intervals (CI) were computed using the Joinpoint regression model to assess internal trends over each independent time period.^12^ This methodology provides a detailed understanding of how rates fluctuate annually, offering a granular perspective on year-to-year changes. The average estimated annual percentage change (EAPC) and its CI were calculated using a log-transformed linear regression model to analyze temporal trends in childhood neuroblastoma incidence, mortality, and DALYs from 1990 to 2021.^13^ The EAPC is especially valuable for examining long-term trends as it reveals whether rates are generally increasing or decreasing over time, regardless of short-term variations. An EAPC value and the lower limit of its 95% CI greater than 0 indicate an increasing trend for the corresponding indicator. Conversely, an EAPC value and the upper limit of its 95% CI less than 0 indicate a decreasing trend for the corresponding indicator. Fitted curves were utilized to analyze the relationship between disease burden indicators and the SDI. All analyses in this study were performed using R language version 4.3.3, with a significance level of p < 0.05.

### Ethics statement

The waiver of the informed consent procedure was approved by the Ethics Committee of Guangxi Medical University.

### Role of the funding source

The funders of the study had no role in study design, data collection, data analysis, data interpretation, or writing of the report. All authors had comprehensive access to all data related to the study and accepted full responsibility for the decision to submit the manuscript for publication.

## Results

### Global burden trends

#### Incidence

**Fig. 1:**
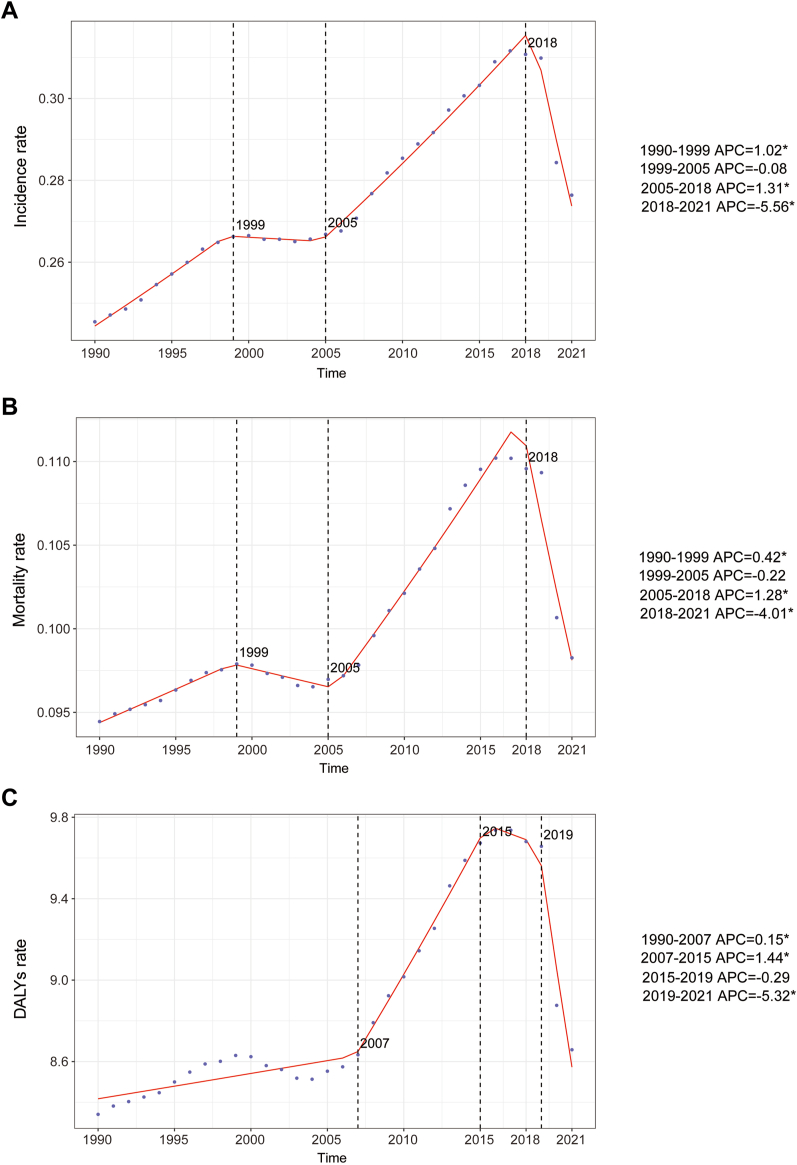
Annual percent change (APC) and trends in global childhood neuroblastoma incidence, mortality, and disability-adjusted life years (DALYs) from 1990 to 2021. A, Incidence rate. B, Mortality rate. C, DALYs rate.

**Fig. 2:**
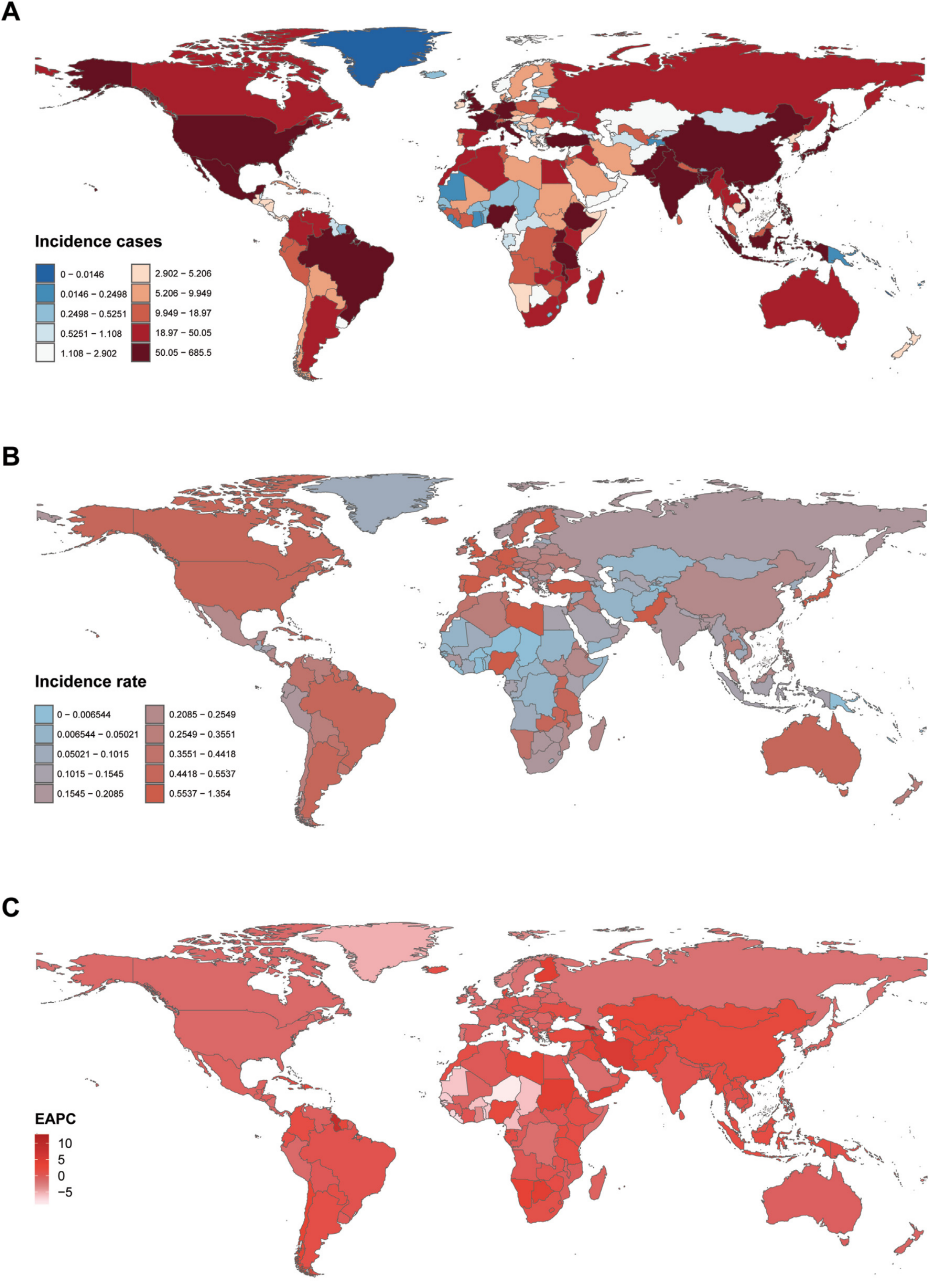
Incidence of childhood neuroblastoma across 204 countries and territories. A, Number of incidence cases. B, Incidence rate. C, Estimated annual percentage change (EAPC) in incidence.

**Fig. 3:**
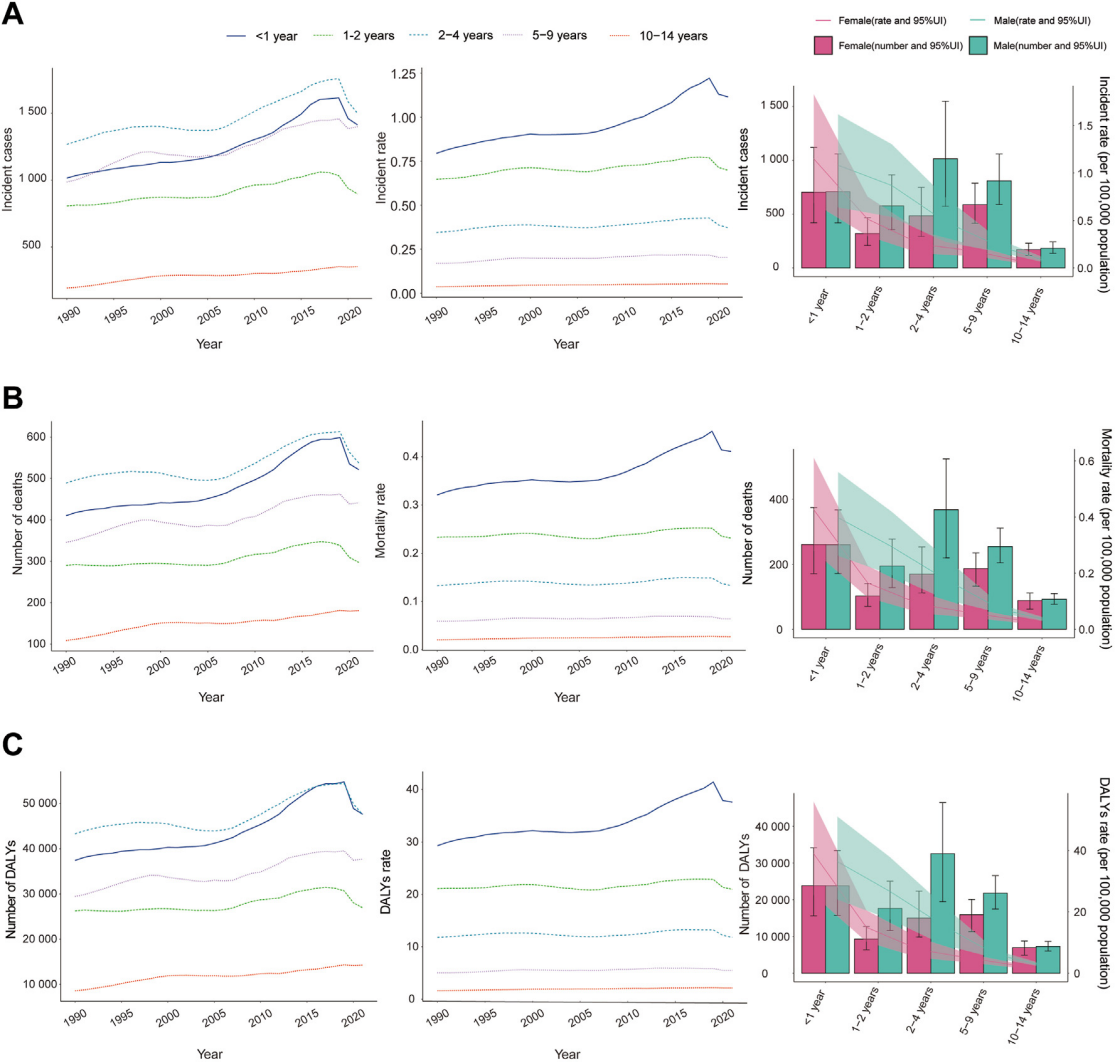
Trends in incidence, mortality, and disability-adjusted life years (DALYs) of childhood neuroblastoma by age and sex, 1990–2021. A, Incidence cases and rates. B, Mortality cases and rates. C, DALYs cases and rates.

**Fig. 4:**
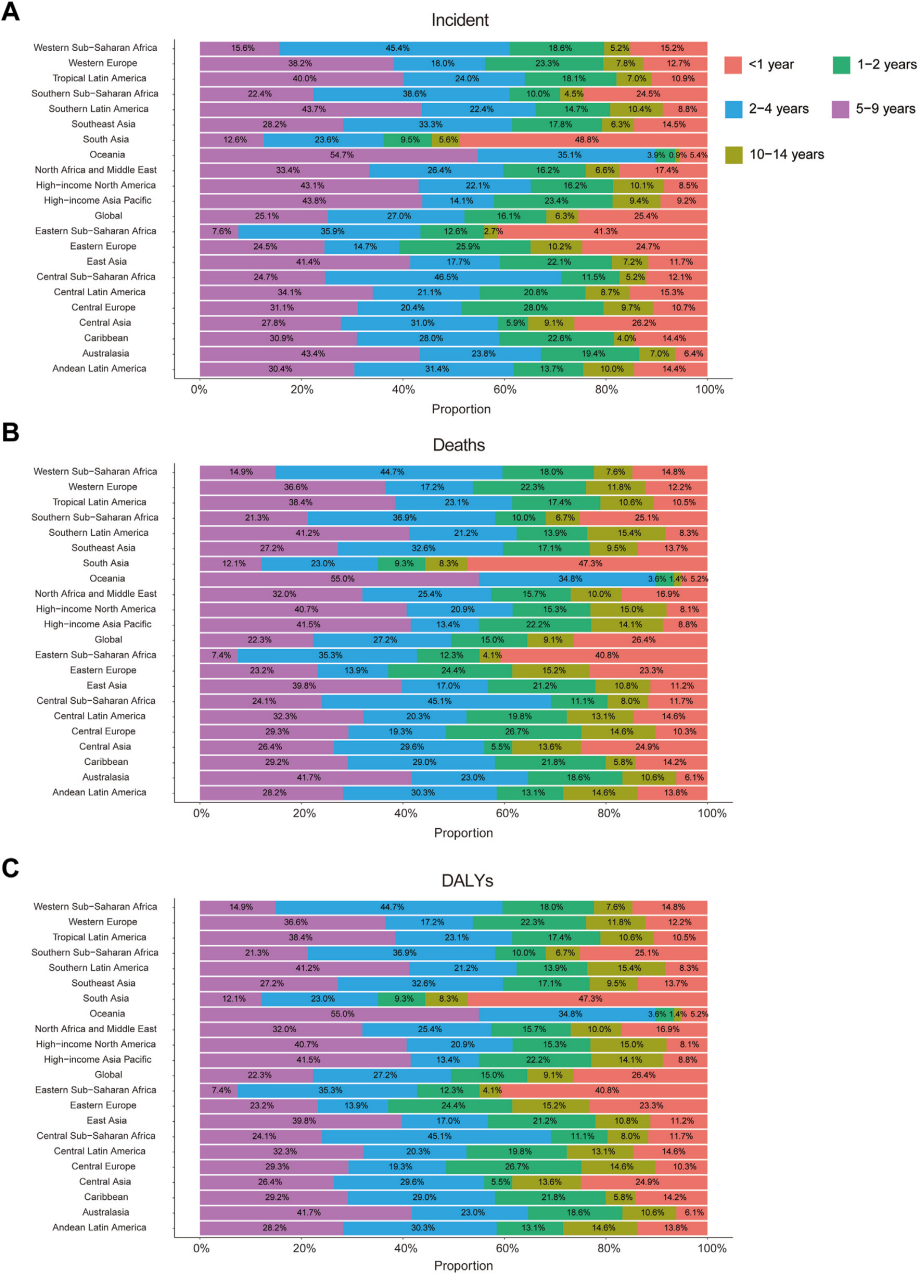
Age-specific percentages of childhood neuroblastoma incidence, mortality, and disability-adjusted life years (DALYs) in 2021. A, Incidence. B, Deaths. C, DALYs.

Large-scale data analysis indicates that the global epidemiologic trend of childhood NB has shown significant dynamics, initially increasing and then decreasing in incidence. The highest APC was observed from 2005 to 2018, with a value of 1.31% (95% CI, 1.22%–1.40%) (Fig. 1A). Furthermore, the peak incidence was observed in 2017, with an incidence rate of 0.31 (95% UI, 0.22–0.41) per 100,000 people (Fig. 1A). In 1990, the global number of NB cases was 4267 (95% UI, 3144.44–5768.06), whereas, in 2021, this number increased to 5560 (95% UI, 3734.21–7560.03), reflecting an overall increase of 30.26% (95% UI, −1.24–72.51%). Similarly, the incidence rate increased from 0.25 (95% UI, 0.18–0.33) per 100,000 people in 1990 to 0.28 (95% UI, 0.19–0.38) per 100,000 people in 2021, an overall increase of 12.60% (95% UI, −14.62%–49.12%). The EAPC was 0.70% (95% CI, 0.58–0.82) (Table 1; Fig. 2). Notably, the incidence of NB increased across all age segments, with the largest increase in children aged 10–14 years (45.87%) and the smallest increase in children aged 2–4 years (7.98%) (Fig. 3A). Children under one year of age exhibited the highest incidence rates of neuroblastoma, comprising 25.4% of all incident cases in 2021, with a corresponding incidence rate of 1.12 per 100,000 people (95% UI, 0.70–1.63). In contrast, children aged 10–14 years consistently demonstrated the lowest incidence, representing only 6.3% of all neuroblastoma incident cases among children in the same year, with an incidence rate of 0.05 per 100,000 people (95% UI, 0.04–0.07) (Figs. 3A and 4A). In terms of gender differences, the incidence rate for girls under 1 year of age was not significantly different from that of boys, whereas it was higher for boys than for girls between the ages of 1 and 14 years, with the most pronounced differences occurring in the 2–4 years age group (Fig. 3A).

**Table 1:** Incidence of neuroblastoma in children between 1990 and 2021 at the global and regional level.

Location	Rate per 100,000 (95% UI)						
	1990		2021		1990–2021		
	Incident cases	Incident rate	Incident cases	Incident rate	Cases change^b^	Rate change^b^	EAPC^a^
Global	4267 (3144.44, 5768.06)	0.25 (0.18, 0.33)	5560 (3734.21, 7560.03)	0.28 (0.19, 0.38)	30.26 (−1.24, 72.51)	12.60 (−14.62, 49.12)	0.70 (0.58, 0.82)
**SDI**							
Low SDI	386 (214.16, 705.53)	0.17 (0.09, 0.31)	1005 (453.29, 1767.58)	0.22 (0.10, 0.38)	160.33 (42.90, 324.38)	29.49 (−28.92, 111.08)	1.09 (0.63, 1.55)
Low–middle SDI	820 (512.80, 1279.65)	0.17 (0.11, 0.27)	1562 (941.88, 2348.29)	0.27 (0.16, 0.40)	90.49 (31.33, 188.65)	55.10 (6.93, 135.02)	1.87 (1.64, 2.10)
Middle SDI	1090 (756.00, 1520.89)	0.19 (0.13, 0.26)	1429 (979.14, 1916.30)	0.25 (0.17, 0.34)	31.04 (−1.27, 80.73)	33.43 (0.53, 84.03)	1.36 (1.12, 1.59)
High–middle SDI	812 (612.99, 1056.23)	0.30 (0.22, 0.39)	739 (544.62, 938.10)	0.32 (0.24, 0.41)	−8.95 (−31.34, 20.15)	7.90 (−18.63, 42.38)	0.94 (0.61, 1.27)
High SDI	1157 (968.87, 1357.28)	0.62 (0.52, 0.73)	822 (679.61, 969.79)	0.48 (0.39, 0.56)	−28.97 (−37.60, 19.08)	−23.51 (−32.80, −12.85)	−0.81 (−1.05, −0.56)
**Regions**							
Andean Latin America	41 (25.14, 62.51)	0.28 (0.17, 0.42)	38 (24.75, 54.41)	0.21 (0.14, 0.30)	−7.09 (−41.55, 49.57)	12.60 (−14.62, 49.12)	−0.40 (−0.63, −0.16)
Australasia	28 (23.28, 34.81)	0.62 (0.51, 0.76)	29 (20.52, 39.60)	0.50 (0.36, 0.69)	1.51 (−26.12, 38.44)	−23.74 (−52.02, 22.77)	−0.53 (−0.78, −0.28)
Caribbean	37 (24.27, 57.14)	0.32 (0.21, 0.50)	55 (35.19, 82.12)	0.48 (0.31, 0.71)	49.05 (5.22, 110.93)	−31.26 (−53.63, −10.46)	1.43 (0.90, 1.95)
Central Asia	13 (8.35, 18.98)	0.05 (0.03, 0.08)	20 (12.60, 29.83)	0.07 (0.05, 0.11)	61.03 (3.25, 147.53)	37.88 (−10.82, 120.29)	2.76 (2.18, 3.34)
Central Europe	95 (70.86, 126.48)	0.32 (0.24, 0.43)	47 (34.88, 59.30)	0.26 (0.20, 0.33)	−51.33 (−64.09, −33.70)	9.93 (−34.70, 90.14)	−0.60 (−1.20, 0.00)
Central Latin America	184 (132.91, 246.61)	0.29 (0.21, 0.38)	162 (113.31, 222.58)	0.25 (0.18, 0.35)	−12.34 (−33.31, 17.90)	16.24 (−42.77, 105.16)	0.20 (−0.85, 0.46)
Central Sub–Saharan Africa	15 (5.73, 31.15)	0.06 (0.02, 0.12)	26 (15.01, 41.91)	0.04 (0.03, 0.07)	73.63 (−4.13, 306.09)	−33.58 (−42.15, 22.17)	−0.60 (−0.98, −0.23)
East Asia	505 (324.05, 766.17)	0.15 (0.10, 0.23)	564 (381.23, 760.08)	0.21 (0.14, 0.28)	11.76 (−27.71, 78.56)	63.73 (6.19, 176.82)	2.07 (1.57, 2.58)
Eastern Europe	143 (102.74, 198.45)	0.28 (0.20, 0.39)	68 (48.73, 90.00)	0.19 (0.14, 0.25)	−52.65 (−68.06, −38.33)	−25.14 (−58.67, 75.07)	−1.00 (−1.16, −0.83)
Eastern Sub–Saharan Africa	243 (135.68, 434.49)	0.27 (0.15, 0.48)	556 (241.12, 992.99)	0.31 (0.14, 0.56)	129.00 (12.76, 304.19)	−11.10 (−32.37, 19.56)	0.84 (0.48, 1.20)
High–income Asia Pacific	245 (200.53, 292.96)	0.69 (0.57, 0.83)	141 (117.83, 165.15)	0.63 (0.53, 0.74)	−42.36 (−52.58, −31.59)	−9.52 (−25.57, 7.37)	−0.75 (−1.13, −0.36)
High–income North America	472 (395.05, 558.44)	0.77 (0.64, 0.91)	334 (273.23, 394.54)	0.51 (0.42, 0.60)	−29.34 (−38.45, −17.19)	47.85 (4.37, 109.23)	−1.08 (−1.28, −0.88)
North Africa and Middle East	247 (151.14, 397.51)	0.18 (0.11, 0.28)	354 (244.00, 500.85)	0.19 (0.13, 0.27)	43.46 (−14.78, 148.12)	−22.12 (−35.37, −5.84)	0.89 (0.57, 1.22)
Oceania	0.28 (0.13, 0.51)	0.01 (0.00, 0.02)	0.46 (0.23, 0.83)	0.01 (0.00, 0.02)	66.48 (−6.30, 199.99)	−18.93 (−40.19, 10.43)	−1.44 (−2.05, −0.83)
South Asia	755 (433.24, 1200.50)	0.17 (0.10, 0.28)	1447 (875.76, 2238.73)	0.29 (0.17, 0.44)	91.56 (24.24, 223.87)	−18.77 (−40.88, 10.77)	2.01 (1.59, 2.43)
Southeast Asia	236 (144.11, 360.46)	0.14 (0.08, 0.21)	329 (229.32, 452.06)	0.19 (0.13, 0.26)	39.66 (0.88, 102.41)	29.59 (−12.75, 96.36)	0.98 (0.86, 1.11)
Southern Latin America	47 (33.16, 64.68)	0.31 (0.22, 0.43)	59 (41.11, 83.87)	0.41 (0.28, 0.58)	25.85 (−15.27, 90.69)	8.23 (−18.33, 42.25)	1.40 (0.96, 1.84)
Southern Sub–Saharan Africa	28 (17.59, 41.44)	0.14 (0.09, 0.20)	43 (27.23, 63.89)	0.18 (0.11, 0.27)	53.71 (2.57, 125.52)	55.48 (−11.23, 129.96)	1.31 (0.86, 1.77)
Tropical Latin America	240 (169.83, 326.91)	0.45 (0.32, 0.61)	243 (170.71, 325.78)	0.48 (0.34, 0.65)	1.32 (−23.55, 33.17)	38.12 (−0.24, 100.18)	0.68 (0.12, 1.24)
Western Europe	522 (439.06, 612.52)	0.74 (0.62, 0.86)	390 (307.12, 486.21)	0.57 (0.45, 0.71)	−25.30 (−38.01, −9.69)	32.14 (−11.82, 93.88)	−0.75 (−1.02, −0.49)
Western Sub–Saharan Africa	173 (48.54, 324.10)	0.20 (0.06, 0.37)	656 (169.18, 1214.70)	0.31 (0.08, 0.57)	279.97 (116.93, 461.97)	45.41 (−6.77, 123.52)	1.84 (1.43, 2.25)

Abbreviations: EAPC, estimated annual percentage change; SDI, Sociodemographic Index; UI, uncertainty interval.

a EAPC is expressed as 95% confidence interval.

b Change shows the percentage change.

#### Mortality

Consistent with morbidity trends, NB-related mortality exhibited an overall increasing and then decreasing trend over the past three decades. The highest APC was recorded from 2005 to 2018, with value of 1.28% (95% CI, 1.14%–1.41%) (Fig. 1B). Furthermore, the peak mortality was observed in 2017, with an mortality rate of 0.11 (95% UI, 0.09–0.13) per 100,000 people (Fig. 1B). The global number of NB deaths in 1990 was 1643 (95% UI, 1373.57–1956.38), while in 2021, the number of deaths was 1977 (95% UI, 1445.04–2528.54), an overall increase of 20.35% (95% UI, −12.44%–63.30%) (Table S1). Similarly, the mortality rate increased from 0.09 (95% UI, 0.08–0.11) per 100,000 people in 1990 to 0.10 (95% UI, 0.07–0.13) per 100,000 people in 2021, an increase of 4.04% (95% UI, −24.31% to 41.16%). The EAPC was 0.49% (95% CI, 0.41–0.56) (Table S1). NB mortality increased in all age segments, with the largest increase in children aged 10–14 years (33.68%) and the smallest increase in children aged 2–4 years (0.23%) (Fig. 3B). Children under one year of age exhibited the highest mortality rate associated with neuroblastoma, accounting for 26.4% of all death cases in 2021, with a corresponding mortality rate of 0.41 per 100,000 people (95% UI, 0.29–0.55). In contrast, children aged 10–14 years consistently demonstrated the lowest mortality, representing only 9.1% of all neuroblastoma death cases among children during the same year, with a mortality rate of 0.03 per 100,000 people (95% UI, 0.02–0.03) (Figs. 3B and 4B). In terms of gender differences, no significant difference was observed between the mortality rates of girls and boys up to the age of 1 year, whereas the mortality rate for boys was higher than that for girls between the ages of 1 and 14 years, with the most pronounced difference in the 2–4 age group (Fig. 3B).

#### DALYs

Consistent with morbidity and mortality trends, the NB-associated DALYs rate exhibited an overall upward and then downward trend over the past three decades. The highest APC was recorded from 2007 to 2015, with value of 1.44% (95% CI, 1.24%–1.65%) (Fig. 1C). Furthermore, the peak DALYs was observed in 2017, with an DALYs rate of 9.74 (95% UI, 7.80–11.58) per 100,000 people (Fig. 1C). The global DALYs for NB in 1990 were 145,057.36 (95% UI, 120,924.76–173,294.30), whereas in 2021, the DALYs increased to 174,186.30 (127,104.64–223,265.92), an overall increase of 20.08% (95% UI, −12.89% to 63.27%) (Table S2). The DALYs rate increased from 8.34 (95% UI, 6.95–9.96) per 100,000 people in 1990 to 8.66 (95% UI, 6.32–11.10) per 100,000 people in 2021, an increase of 3.80% (95% UI, −24.69% to 41.14%). The EAPC was 0.45% (95% CI, 0.32%–0.57%) (Table S2). The number of DALYs increased across all age segments, with the largest increase in children aged 10–14 years (66.57%) and the smallest increase in children aged 2–4 years (9.82%) (Fig. 3C). Children under one year of age exhibited the highest DALYs rate associated with neuroblastoma, accounting for 26.4% of all DALYs cases in 2021, with a corresponding DALYs rate of 37.60 per 100,000 people (95% UI, 0.29–0.55). In contrast, children aged 10–14 years consistently demonstrated the lowest DALYs rate, representing only 9.1% of all neuroblastoma DALYs cases among children during the same year, with a DALYs rate of 2.14 per 100,000 individuals (95% UI, 1.69–2.47) (Figs. 3C and 4C). Gender differences were also present in the number of DALYs, with no significant difference between the number of DALYs for girls and boys under 1 year of age. However, the number of DALYs was higher for boys than for girls between the ages of 1 and 14 years, with the most pronounced difference in the 2–4 years age group (Fig. 3C).

### Neuroblastoma in children: regional trends by SDI

**Fig. 5:**
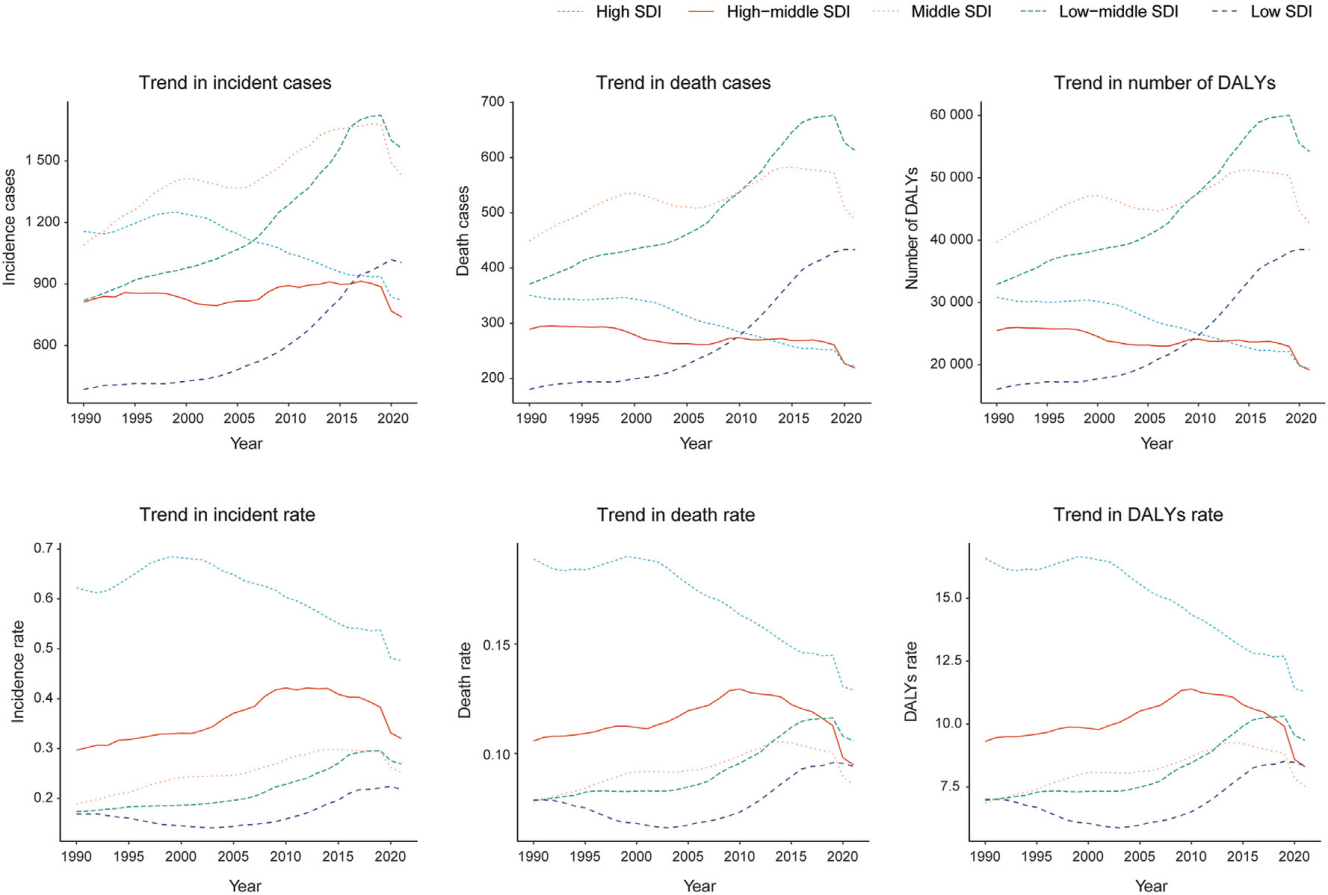
Epidemiologic trends in childhood neuroblastoma incidence, mortality, and disability-adjusted life years (DALYs) rates across five Sociodemographic Index (SDI) areas from 1990 to 2021.

**Fig. 6:**
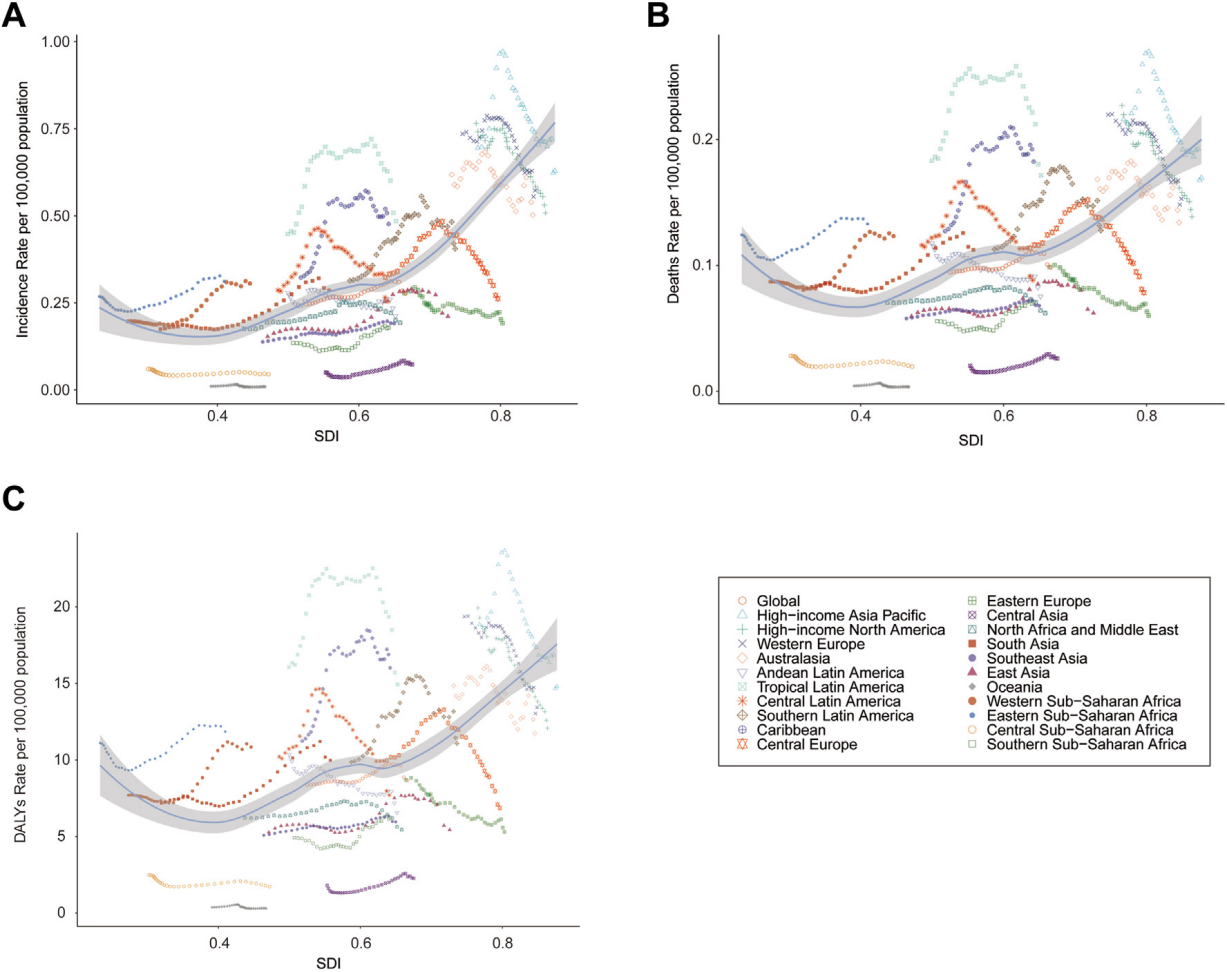
Association between incidence, mortality, and disability-adjusted life years (DALYs) rates of childhood neuroblastoma and regional Sociodemographic Index (SDI), 1990–2021. A, Incidence rate. B, Mortality rate. C, DALYs rates.

Compared to 1990, the incidence, mortality, and DALYs in low SDI, low-intermediate SDI regions, and intermediate SDI regions showed an increasing trend in 2021. The largest increases were observed in the number of incidence cases (1562 cases; 95% UI, 941.88–2348.29 cases), deaths (613 cases; 95% UI, 433.07–819.67 cases), and DALYs (54,242.65; 95% UI, 38,164.86–72,461.29) (Table 1; Tables S1 and S2; Fig. 5). Moreover, the incidence-associated EAPC (1.87%; 95% CI, 1.64–2.10), mortality-associated EAPC (1.22%; 95% CI, 1.09–1.34), and DALYs-associated EAPC (1.36%; 95% CI, 1.15–1.57) of childhood neuroblastoma were highest in the low-middle SDI region (Table 1; Tables S1 and S2; Fig. 5). In contrast, the incidence, mortality, and DALYs decreased in both medium–high SDI and high SDI regions, especially in high SDI regions where pediatric neuroblastoma incidence-associated EAPC (−0.81%; 95% CI, −1.05%, −0.56%), mortality-associated EAPC (−0.93%; 95% CI, −1.04%, −0.81%), and DALYs-associated EAPC (−1.14%; 95% CI, −1.32%, −0.96%) were the lowest (Table 1; Tables S1 and S2; Fig. 5, Fig. 6).

### Neuroblastoma in children: national trends

#### Incidence

In 2021, India had the highest number of neuroblastoma cases worldwide (685 cases; 95% UI, 404.16–1007.67), while Malta had the highest incidence rate (1.35 per 100,000 people; 95% UI, 0.86–2.00) (Table S3; Fig. 2A–B). From 1990 to 2021, Georgia exhibited the largest increase in incidence (EAPC = 12.66%; 95% CI, 11.11%–14.22%), whereas Niger showed the largest decrease (EAPC = −8.81%; 95% CI, −9.56% to −8.06%) (Table S3; Fig. 2C). The global incidence of neuroblastoma was 0.28 (95% UI, 0.19–0.38) per 100,000 people in 2021, higher than the global incidence in 77 of 204 countries and lower than the global incidence in 127 countries.

#### Mortality

In 2021, Pakistan had the highest number of deaths from neuroblastoma and other peripheral nerve cell tumors globally (268 cases; 95% UI, 165.09–426.35), while Malawi had the highest mortality rate (0.42 per 100,000 people; 95% UI, 0.16–0.87) (Table S4; Figure S1A–B). From 1990 to 2021, Georgia exhibited the largest increase in mortality (EAPC = 12.57%; 95% CI, 11.03–14.13), whereas Niger showed the largest decrease (EAPC = −8.71%; 95% CI, −9.46 to −7.97) (Table S4; Figure S1C). The global mortality rate of neuroblastoma and other peripheral nerve cell tumors was 0.10 (95% UI, 0.07–0.13) per 100,000 people in 2021, higher than the global rate in 67 of 204 countries and lower than the global rate in 137 countries.

#### DALYs

In 2021, Pakistan had the highest number of DALYs for neuroblastoma and other peripheral nerve cell tumors globally (23,878.68; 95% UI, 14,590.73–37,924.28), while Malawi had the highest rate of DALYs (37.34 per 100,000 people; 95% UI, 14.04–78.41) (Table S5; Figure S2A–B). From 1990 to 2021, Georgia exhibited the largest increase in DALYs (EAPC = 12.6%; 95% CI, 11.05%–14.18%), whereas Niger showed the largest decrease (EAPC = −8.78%; 95% CI, −9.52% to −8.03%) (Table S5; Figure S2C). The global DALYs rate of neuroblastoma and other peripheral nerve cell tumors was 8.66 (95% UI, 6.32–11.10) per 100,000 people in 2021, higher than the global DALYs rate in 70 of 204 countries and lower than the global DALYs rate in 134 countries.

## Discussion

Neuroblastoma, the most common extracerebral solid tumor in children worldwide, presents a high mortality rate that profoundly impacts children's lives and mental health, compromises families' well-being, and escalates medical and social costs.^14^ A comprehensive investigation of neuroblastoma incidence, mortality, and DALYs is crucial for formulating effective public health strategies. However, there has been no global-scale statistical analysis of the epidemiology of childhood neuroblastoma across various regions and countries. Previous analyses have been limited to specific countries or regions. This study is the first to utilize GBD data from 1990 to 2021 to analyze the incidence, mortality, and DALYs of childhood neuroblastoma globally, segmented by region, country, sex, and age, thereby providing reliable data for future public health policymakers and facilitating the development of targeted prevention and control strategies.

The results of this study indicate that from 1990 to 2021, the global incidence, mortality, and DALYs of childhood neuroblastoma initially increased, then decreased, but overall demonstrated a rising trend, with percentage changes of 12.60%, 4.04%, and 3.80%, respectively. The lower mortality compared to morbidity in neuroblastoma can be attributed to over three decades of advancements in treatment strategies developed by clinicians and researchers. Recent studies indicate that survival rates for patients with high-risk neuroblastoma have improved significantly, increasing from 10 to 20% in the 1990s to over 50% today.^15^^,^^16^ The overall mortality figures presented in this report represent data aggregated for all neuroblastoma patients, irrespective of their risk classification. Consequently, these general mortality statistics may obscure notable progress made in specific subgroups, such as patients diagnosed with high-risk (HR) neuroblastoma. Although substantial advancements have been documented in the survival rates for HR neuroblastoma, these improvements may not have been universally accessible or adopted in regions with lower SDI. Disparities in healthcare infrastructure, limited access to advanced therapeutic options—such as immunotherapy and stem cell transplantation—and inadequate follow-up care likely contribute to varying outcomes between countries and regions.^17^ These factors may, in turn, affect the global mortality data for neuroblastoma. Moreover, the observed global incidence rate, reflected by an EAPC of 0.70%, suggests that the modest increase in incidence could be linked to several factors. Advances in early detection technologies, enhanced screening protocols, and heightened clinical awareness likely contribute to this trend. Specifically, improvements in imaging modalities, such as PET/CT, have greatly enhanced the ability to detect tumors at earlier stages, including smaller or asymptomatic ones that might previously have gone undiagnosed until they had progressed to more advanced stages. The introduction of biomarkers, such as urinary catecholamine metabolites homovanillic acid (HVA) and vanillylmandelic acid (VMA),^18^ has also provided cost-effective, non-invasive screening options. Additionally, increased clinical awareness of neuroblastoma among pediatricians, oncologists, and general practitioners has resulted in greater vigilance for early signs of the disease, particularly in patients presenting with non-specific or ambiguous symptoms. Notably, our study suggests a notable decline in neuroblastoma incidence and mortality from 2018 to 2021, likely linked to the COVID-19 pandemic. The pandemic caused significant disruptions to global health services, including delays in cancer diagnoses, reduced access to non-emergency care, and interruptions to routine screening programs. These factors may have temporarily lowered reported neuroblastoma cases, as patients and families avoided healthcare facilities due to fears of COVID-19 exposure.^19^^,^^20^

Trends in neuroblastoma incidence and mortality exhibit distinct patterns across different SDI regions. Specifically, a decreasing trend in neuroblastoma incidence and mortality are observed in high SDI and medium–high SDI regions, while an increasing trend is noted in medium, medium-low, and low SDI regions. In high SDI regions, a steady decline in both incidence and mortality rates can be attributed to significant advances in early detection, improved screening programs, and heightened clinical awareness. The implementation of comprehensive cancer screening initiatives, coupled with the availability of advanced diagnostic tools, has facilitated earlier diagnosis, thereby enabling more effective therapeutic interventions. In addition, the adoption of multimodal therapies—including chemotherapy, radiotherapy, and surgical interventions—has been instrumental in reducing mortality rates associated with neuroblastoma.^21^^,^^22^ In contrast, medium, low-middle, and low SDI regions are experiencing a growing burden of neuroblastoma, primarily due to insufficient healthcare infrastructure and limited access to early diagnostic tools. Additionally, the gradual decline in birth rates in high SDI and medium–high SDI regions, juxtaposed with significantly higher birth rates in medium, medium-low, and low SDI regions, contributes substantially to the observed disparities in neuroblastoma incidence and mortality. This demographic shift further emphasizes the need for targeted public health interventions to enhance early detection and treatment accessibility in lower SDI regions.

In 2021, approximately 70% of all neuroblastoma cases occur in children under five years of age. This high incidence is related to the origin of neuroblastoma from neural crest cells involved in the development of the sympathetic nervous system.^23^ These cells are most active during fetal development and early childhood, making this period most susceptible to oncogenic mutations and transformations. Regarding gender, the incidence, mortality, and DALYs rates of neuroblastoma are higher in males than females, with the most pronounced gender difference observed in the 2–4 years age group, where the incidence and mortality ratios of boys to girls are 1.96:1 and 2.02:1, respectively. This may be due to sex chromosome differences contributing to cancer susceptibility.^24^ Additionally, neuroblastoma in males often has a worse prognosis, possibly linked to metastasis.^25^ The gender-specific bias in cancer prognosis and tumor biological behavior is not exclusive to neuroblastoma. Similar trends are observed in various other cancers, where male patients often exhibit more severe outcomes.^26^

Previous studies have revealed the heterogeneity of genomic abnormalities and molecular mechanisms underlying neuroblastoma from genetic, transcriptional, and hereditary perspectives.^27^ However, the pathogenesis of neuroblastoma remains largely unknown. At the genetic level, the most extensively studied genetic alteration in neuroblastoma is MYCN amplification, which, even in the absence of other significant adverse prognostic factors, is sufficient to categorize patients as high-risk. Nevertheless, there is no direct evidence demonstrating that MYCN causes neuroblastoma.^28^ ALK (anaplastic lymphoma kinase) mutations have also been identified as drivers of tumor development in both familial and sporadic cases of neuroblastoma. Furthermore, structural chromosomal abnormalities, such as deletions of 1p and 11, as well as gains in 17q, are key indicators of poor prognosis in neuroblastoma.^29^ From a molecular standpoint, aberrant expression of genes such as MYCN, LIN28B, PHOX2B, and ALK contributes to the proliferation and differentiation of neuroblastoma cells, driving tumorigenesis and promoting tumor progression.^30^, ^31^, ^32^ Studies have shown that neuroblastoma consists of two epigenetic identities: adrenergic (ADRN) and mesenchymal (MES), which can interconvert. This plasticity and intra-tumoral heterogeneity are driven by transcriptional and epigenetic reprogramming, further complicating the disease's clinical management.^33^ Advances in genomic technologies have provided deeper insights into the molecular basis of neuroblastoma, highlighting potential therapeutic targets. However, additional research is required to fully elucidate the complex mechanisms driving neuroblastoma and to develop more effective treatment strategies. Looking ahead, it is anticipated that further research will shed new light on the complex pathogenesis of neuroblastoma, which is crucial for formulating novel preventive and therapeutic strategies for treating pediatric neuroblastoma.

In conclusion, from 1990 to 2021, the global incidence, mortality, and DALYs of childhood neuroblastoma initially increased, then decreased, but generally exhibited an upward trend. The disease burden continues to rise in low, low-medium, and medium SDI regions. Consequently, policymakers must urgently develop more effective prevention and control measures to reduce the disease burden of childhood neuroblastoma, improve family well-being, and alleviate economic pressures on society.

This study, being the first to use an epidemiological model to analyze the global disease burden of childhood neuroblastoma, provides valuable insights for countries and healthcare professionals to develop reasonable preventive measures, management policies, and diagnostic and treatment protocols. However, several limitations exist. Firstly, although neuroblastoma predominantly affects children aged 0–14 years, the inclusion of other peripheral nerve tumors in the statistical analysis may introduce potential inaccuracies in the results. Second, as a cross-sectional study based on the GBD database, the data source and accuracy are influenced by different national statistical agencies and health departments, including variations in definitions and methodological inconsistencies. Such discrepancies could result in an underestimation of neuroblastoma incidence. Third, data availability in less developed regions is concerning, as a large number of undiagnosed cases of childhood neuroblastoma may not accurately reflect the true disease burden. Finally, the GBD database does not offer detailed risk stratification between localized and metastatic neuroblastoma cases, which limits the ability to conduct subgroup analyses. This restriction hinders the evaluation of disease outcomes based on severity, thereby impacting the study's capacity to provide more nuanced insights into neuroblastoma prognosis and treatment efficacy.

## Contributors

BS and YGL conceptualized the study. JSN, CS, and CHL developed the study protocol. CJW, WL, YL, PC, YQL, and ZHL conducted the analysis of the GBD data. CC and QL were responsible for the statistical analysis and interpretation of the data. XJS, ZXY, and STL performed the literature search. JSN drafted the manuscript, which was critically revised by the other authors. JSN and BS accessed and verified the data. All authors reviewed and approved the final version of the manuscript.

## Data sharing statement

The data and codes utilized or analyzed in this study are accessible from the corresponding author upon request.

## Editor note

The Lancet Group takes a neutral position with respect to territorial claims in published maps and institutional affiliations.

## Declaration of interests

The authors declare no conflict of interest.

## References

[1] Matthay K.K., Villablanca J.G., Seeger R.C. (1999). Treatment of high-risk neuroblastoma with intensive chemotherapy, radiotherapy, autologous bone marrow transplantation, and 13-cis-retinoic acid. Children's Cancer Group. N Engl J Med.

[2] Maris J.M., Hogarty M.D., Bagatell R., Cohn S.L. (2007). Neuroblastoma. Lancet (London, England).

[3] Gatta G., Botta L., Rossi S. (2014). Childhood cancer survival in Europe 1999-2007: results of EUROCARE-5--a population-based study. Lancet Oncol.

[4] Jansky S., Sharma A.K., Körber V. (2021). Single-cell transcriptomic analyses provide insights into the developmental origins of neuroblastoma. Nat Genet.

[5] Körber V., Stainczyk S.A., Kurilov R. (2023). Neuroblastoma arises in early fetal development and its evolutionary duration predicts outcome. Nat Genet.

[6] Gurney J.G., Swensen A.R., Bulterys M., Ries L.G., Smith M.J.N.P. (1999).

[7] Stiller C.A., Parkin D.M. (1992). International variations in the incidence of neuroblastoma. Int J Cancer.

[8] (2024). Global burden of 288 causes of death and life expectancy decomposition in 204 countries and territories and 811 subnational locations, 1990-2021: a systematic analysis for the Global Burden of Disease Study 2021. Lancet (London, England).

[9] (2024). Global incidence, prevalence, years lived with disability (YLDs), disability-adjusted life-years (DALYs), and healthy life expectancy (HALE) for 371 diseases and injuries in 204 countries and territories and 811 subnational locations, 1990-2021: a systematic analysis for the Global Burden of Disease Study 2021. Lancet (London, England).

[10] von Elm E., Altman D.G., Egger M., Pocock S.J., Gøtzsche P.C., Vandenbroucke J.P. (2007). The Strengthening the Reporting of Observational Studies in Epidemiology (STROBE) statement: guidelines for reporting observational studies. Lancet (London, England).

[11] Kocarnik J.M., Compton K., Dean F.E. (2022). Cancer incidence, mortality, years of life Lost, years lived with disability, and disability-adjusted life years for 29 cancer groups from 2010 to 2019: a systematic analysis for the global burden of disease study 2019. JAMA Oncol.

[12] Kim H.J., Fay M.P., Feuer E.J., Midthune D.N. (2000). Permutation tests for joinpoint regression with applications to cancer rates. Stat Med.

[13] Cao G., Liu J., Liu M. (2022). Global, regional, and national incidence and mortality of neonatal preterm birth, 1990-2019. JAMA Pediatr.

[14] Smith M.A., Seibel N.L., Altekruse S.F. (2010). Outcomes for children and adolescents with cancer: challenges for the twenty-first century. J Clin Oncol.

[15] Qiu B., Matthay K.K. (2022). Advancing therapy for neuroblastoma. Nat Rev Clin Oncol.

[16] Pinto N.R., Applebaum M.A., Volchenboum S.L. (2015). Advances in risk classification and treatment strategies for neuroblastoma. J Clin Oncol.

[17] Ladenstein R., Pötschger U., Valteau-Couanet D. (2018). Interleukin 2 with anti-GD2 antibody ch14.18/CHO (dinutuximab beta) in patients with high-risk neuroblastoma (HR-NBL1/SIOPEN): a multicentre, randomised, phase 3 trial. Lancet Oncol.

[18] Amano H., Uchida H., Harada K. (2024). Scoring system for diagnosis and pretreatment risk assessment of neuroblastoma using urinary biomarker combinations. Cancer Sci.

[19] Nogueira L.M., Palis B., Boffa D., Lum S., Yabroff K.R., Nelson H. (2023). Evaluation of the impact of the COVID-19 pandemic on reliability of cancer surveillance data in the national cancer database. Ann Surg Oncol.

[20] Negoita S., Chen H.S., Sanchez P.V. (2024). Annual Report to the Nation on the Status of Cancer, part 2: early assessment of the COVID-19 pandemic's impact on cancer diagnosis. Cancer.

[21] Pritchard-Jones K., Pieters R., Reaman G.H. (2013). Sustaining innovation and improvement in the treatment of childhood cancer: lessons from high-income countries. Lancet Oncol.

[22] Vollset S.E., Goren E., Yuan C.W. (2020). Fertility, mortality, migration, and population scenarios for 195 countries and territories from 2017 to 2100: a forecasting analysis for the Global Burden of Disease Study. Lancet.

[23] Tsubota S., Kadomatsu K. (2018). Origin and initiation mechanisms of neuroblastoma. Cell Tissue Res.

[24] Djos A., Svensson J., Gaarder J. (2024). Loss of chromosome Y in neuroblastoma is associated with high-risk disease, 11q-deletion, and telomere maintenance. Gene Chromosome Cancer.

[25] Liu S., Yin W., Lin Y. (2023). Metastasis pattern and prognosis in children with neuroblastoma. World J Surg Oncol.

[26] Rubin J.B., Lagas J.S., Broestl L. (2020). Sex differences in cancer mechanisms. Biol Sex Differ.

[27] Schwab M., Westermann F., Hero B., Berthold F. (2003). Neuroblastoma: biology and molecular and chromosomal pathology. Lancet Oncol.

[28] Corbacioglu S., Lode H., Ellinger S. (2024). Irinotecan and temozolomide in combination with dasatinib and rapamycin versus irinotecan and temozolomide for patients with relapsed or refractory neuroblastoma (RIST-rNB-2011): a multicentre, open-label, randomised, controlled, phase 2 trial. Lancet Oncol.

[29] Schleiermacher G., Bourdeaut F., Combaret V. (2005). Stepwise occurrence of a complex unbalanced translocation in neuroblastoma leading to insertion of a telomere sequence and late chromosome 17q gain. Oncogene.

[30] Liu Y., Zhang J., Cao F. (2023). N6-methyladenosine-mediated overexpression of long noncoding RNA ADAMTS9-AS2 triggers neuroblastoma differentiation via regulating LIN28B/let-7/MYCN signaling. JCI Insight.

[31] Cardani S., Di Lascio S., Belperio D. (2018). Desogestrel down-regulates PHOX2B and its target genes in progesterone responsive neuroblastoma cells. Exp Cell Res.

[32] Lopez-Delisle L., Pierre-Eugène C., Louis-Brennetot C. (2018). Activated ALK signals through the ERK-ETV5-RET pathway to drive neuroblastoma oncogenesis. Oncogene.

[33] Ponzoni M., Bachetti T., Corrias M.V. (2022). Recent advances in the developmental origin of neuroblastoma: an overview. J Exp Clin Cancer Res.

