# Supplementary material for: Global, regional, and national epidemiology of childhood neuroblastoma (1990–2021): a statistical analysis of incidence, mortality, and DALYs
Source: eClinicalMedicine. 2024 Dec 6;79:102964. doi: 10.1016/j.eclinm.2024.102964 (PMC11667623; doi:10.1016/j.eclinm.2024.102964)
Supplement: Supplementary Figures and Tables [file mmc1.pdf]

Figure S1. Mortality from childhood neuroblastoma in 204 countries and territories. A, Number of deaths. B, Mortality rates. C, Estimated annual percentage change (EAPC) in Mortality.

Figure S2. Disability-adjusted life years (DALYs) for childhood neuroblastoma in 204 countries and territories. A, Number of DALYs cases. B, DALYs rates. C, Estimated annual percentage change (EAPC) in DALYs.

Table S1. Mortality of Neuroblastoma in Children Between 1990 and 2021 at the Global and Regional Level.

Table S2. Disability-adjusted life years (DALYs) of Neuroblastoma in Children Between 1990 and 2021 at the Global and Regional Level.

Table S3. Incidence of neuroblastoma in children, by countries and territories, 1990-2021.

Table S4. Mortality of neuroblastoma in children, by countries and territories, 1990-2021.

Table S5. Disability-adjusted life years (DALYs) of neuroblastoma in children, by countries and territories, 1990-2021.

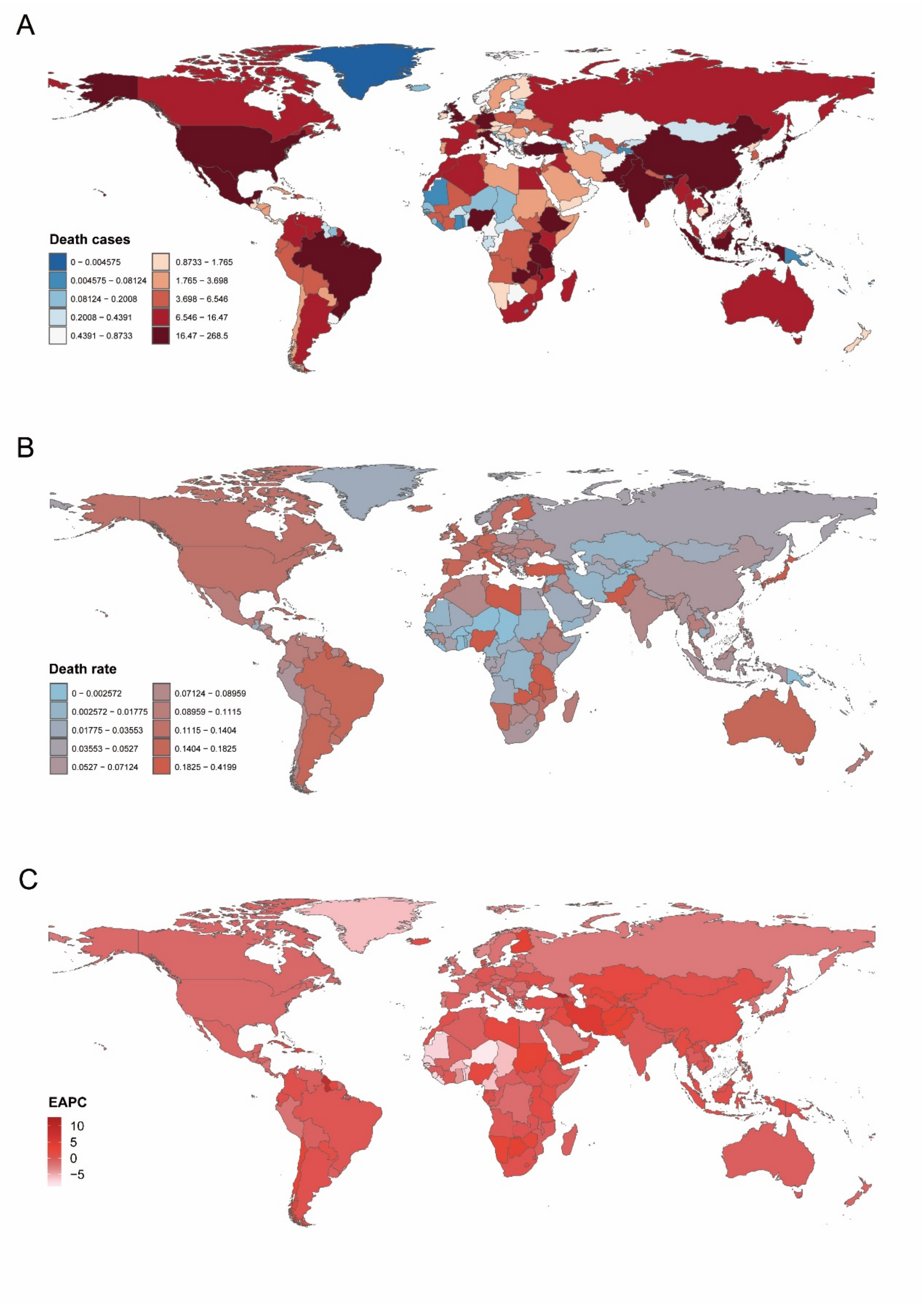

Figure S1. Mortality from childhood neuroblastoma in 204 countries and territories. A, Number of deaths. B, Mortality rates. C, Estimated annual percentage change (EAPC) in Mortality.

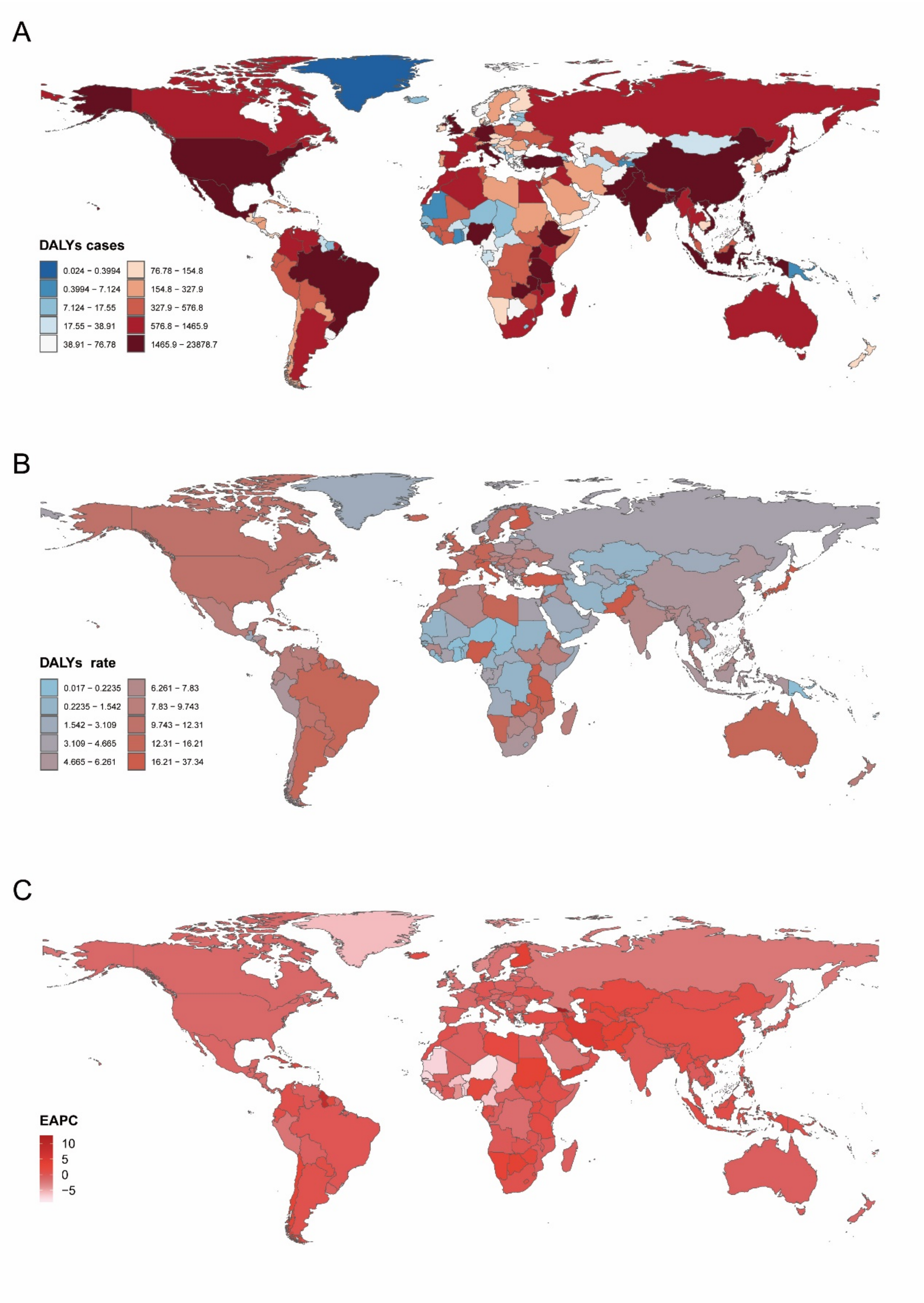

Figure S2. Disability-adjusted life years (DALYs) for childhood neuroblastoma in 204 countries and territories. A, Number of DALYs cases. B, DALYs rates. C, Estimated annual percentage change (EAPC) in DALYs.

Table S1. Mortality of Neuroblastoma in Children Between 1990 and 2021 at the Global and Regional Level.

	Rate per 100 000(95%UI)						
	**1990**		**2021**		**1990-2021**		
**location**	**Number of deaths**	**Mortality rate**	**Number of deaths**	**Mortality rate**	**Cases change[b]**	**Rate change[b]**	**EAPC[a]**
Global	1643(1373.57, 1956.38)	0.09(0.08, 0.11)	1977(1445.04, 2528.54)	0.10(0.07, 0.13)	20.35(-12.44 to 63.30)	4.04(-24.31, 41.16)	0.49(0.41, 0.56)
**SDI**							
Low SDI	180 (120.54, 281.48)	0.08(0.05, 0.12)	433 (234.36, 696.99)	0.09(0.05, 0.15)	140.39(32.14 to 294.44)	19.57(-34.27, 96.19)	0.39(0.13, 0.64)
Low - middle SDI	371 (280.76, 493.61)	0.08(0.06, 0.10)	613 (433.07, 819.67)	0.11(0.07, 0.14)	65.26(11.71 to 151.07)	34.56(-9.04, 104.43)	1.22(1.09, 1.34)
Middle SDI	450(365.93, 527.86)	0.08(0.06, 0.09)	487 (363.58, 614.45)	0.09(0.06, 0.11)	8.29(-17.96 to 45.39)	10.27(-16.47, 48.04)	1.14(0.97, 1.32)
High - middle SDI	289 (245.25, 337.63)	0.11(0.09, 0.12)	219 (170.19, 264.65)	0.09(0.07, 0.11)	-24.25(-42.35 to -0.12)	-10.23(-31.68, 18.37)	0.57(0.37, 0.76)
High SDI	351(332.26, 368.33)	0.19(0.18, 0.20)	223(195.96, 249.04)	0.13(0.11, 0.14)	-36.52(-43.38 to -29.51)	-31.64(-39.02, -24.09)	-0.93(-1.04, -0.81)
**Regions**							
Andean Latin America	17 (12.38, 23.09)	0.12(0.08, 0.16)	14(9.58, 18.29)	0.08(0.05, 0.10)	-22.13(-51.00 to 20.89)	-36.08(-59.78, -0.78)	-1.07(-1.18, -0.96)

Australasia	8(6.69, 8.90)	0.17(0.15, 0.19)	8(5.70, 10.27)	0.13(0.10, 0.18)	-0.39(-27.50 to 34.92)	-20.29(-41.99, 7.96)	-0.54(-0.70, -0.39)
Caribbean	15(10.71, 20.53)	0.13(0.09, 0.18)	21(14.59, 30.27)	0.18(0.13, 0.26)	44.60(1.78 to 104.45)	43.43(0.96, 102.80)	1.67(1.41, 1.92)
Central Asia	5 (3.83, 6.82)	0.02(0.02, 0.03)	7 (5.07, 10.18)	0.03(0.02, 0.04)	41.11(-9.44 to 115.52)	27.42(-18.22, 94.62)	0.54(-0.01, 1.09)
Central Europe	34(28.23, 40.12)	0.11(0.10, 0.14)	14(11.24, 17.16)	0.08(0.06, 0.10)	-58.64(-69.45 to -43.86)	-31.11(-49.11, -6.50)	-0.17(-0.58, 0.25)
Central Latin America	75(68.18, 82.53)	0.12(0.11, 0.13)	56 (43.28, 72.57)	0.09(0.07, 0.11)	-25.06(-43.11 to -2.85)	-24.01(-42.30, -1.48)	0.28(-0.15, 0.72)
Central Sub - Saharan Africa	7 (2.91, 13.60)	0.03(0.01, 0.05)	12(7.84, 16.83)	0.02(0.01, 0.03)	62.18(-10.99 to 271.34)	-30.08(-61.63, 60.09)	-0.76(-0.98, -0.54)
East Asia	197(144.66, 259.99)	0.06(0.04, 0.08)	166(119.26, 217.09)	0.06(0.04, 0.08)	-15.48(-42.98 to 28.82)	4.27(-29.66, 58.93)	1.80(1.44, 2.16)
Eastern Europe	51(40.70, 66.03)	0.10(0.08, 0.13)	21 (16.35, 25.94)	0.06(0.05, 0.07)	-57.99(-71.16 to -46.84)	-39.01(-58.13, -22.82)	-1.36(-1.46, -1.27)
Eastern Sub - Saharan Africa	113(77.61, 187.76)	0.12(0.09, 0.21)	237 (118.02, 394.53)	0.13(0.07, 0.22)	110.90(4.75 to 275.74)	7.05(-46.83, 90.72)	0.21(0.00, 0.42)
High - income Asia Pacific	76 (68.28, 86.50)	0.22(0.19, 0.25)	38 (33.68, 42.90)	0.17(0.15, 0.19)	-50.00(-58.59 to -41.21)	-21.52(-35.00, -7.72)	-0.72(-0.93, -0.50)
High - income North America	140 (129.33, 149.86)	0.23(0.21, 0.24)	91 (78.84, 104.02)	0.14(0.12, 0.16)	-34.99(-44.10 to -25.88)	-38.90(-47.46, -30.34)	-1.39(-1.48, -1.29)
North Africa and Middle East	99 (69.43, 142.20)	0.07(0.05, 0.10)	114(82.47, 151.78)	0.06(0.04, 0.08)	15.04(-30.99 to 95.02)	-11.84(-47.12, 49.45)	0.27(0.11, 0.44)
Oceania	0.11(0.06, 0.20)	0.00(0.00, 0.01)	0.18(0.10, 0.30)	0.00(0.00, 0.01)	54.28(-14.97 to 180.16)	-18.63(-55.15, 47.77)	-0.51(-0.98, -0.04)

South Asia	349 (235.49, 482.28)	0.08(0.05, 0.11)	570(388.20, 802.74)	0.11(0.08, 0.16)	63.12(3.22 to 169.55)	39.42(-11.78, 130.39)	1.13(0.91, 1.34)
Southeast Asia	99(69.57, 136.78)	0.06(0.04, 0.08)	117 (90.75, 149.20)	0.07(0.05, 0.09)	18.92(-13.36 to 71.71)	17.60(-14.32, 69.82)	0.82(0.72, 0.93)
Southern Latin America	17(13.09, 22.32)	0.11(0.09, 0.15)	19(13.29, 25.41)	0.13(0.09, 0.18)	10.05(-25.69 to 63.58)	13.32(-23.48, 68.45)	1.03(0.74, 1.32)
Southern Sub - Saharan Africa	12(8.60, 15.54)	0.06(0.04, 0.08)	17 (12.76, 22.77)	0.07(0.05, 0.09)	47.85(-2.24 to 115.38)	27.10(-15.95, 85.17)	0.76(0.52, 1.00)
Tropical Latin America	98 (84.24, 112.25)	0.18(0.16, 0.21)	86 (65.95, 106.08)	0.17(0.13, 0.21)	-12.31(-32.76 to 12.27)	-6.33(-28.17, 19.92)	0.83(0.50, 1.16)
Western Europe	156 (148.72, 164.27)	0.22(0.21, 0.23)	104 (87.93, 123.65)	0.15(0.13, 0.18)	-33.22(-44.03 to 20.34)	-30.38(-41.65, -16.95)	-0.70(-0.87, -0.53)
Western Sub - Saharan Africa	76(21.62, 120.16)	0.09(0.02, 0.14)	265(66.82, 462.49)	0.12(0.03, 0.22)	246.11(97.98 to 399.66)	41.63(-18.99, 104.46)	1.04(0.78, 1.29)

Abbreviations: EAPC, estimated annual percentage change; SDI, Sociodemographic Index; UI, uncertainty interval.

[a] EAPC is expressed as 95% confidence interval. [b] Change shows the percentage change.

Table S2. DALYs of Neuroblastoma in Children Between 1990 and 2021 at the Global and Regional Level.

	Rate per 100 000(95%UI)						
	1990		2021		1990-2021		
**location**	**Number of DALYs**	**DALYs rate**	**Number of DALYs**	**DALYs rate**	**Cases change** [b]	**Rate change** [b]	**EAPC** [a]
Global	145057.36(120924.76-173294.3)	8.34(6.95-9.96)	174186.30(127104.64-223265.92)	8.66(6.32-11.10)	20.08(-12.89-63.27)	3.80(-24.69-41.14)	0.45(0.32-0.57)
**SDI**							
Low SDI	16054.22(10755.50-24982.99)	7.01(4.70-10.91)	38449.70(20758.04-61655.36)	8.35(4.51-13.40)	139.50(31.59-293.40)	19.13(-34.55-95.67)	0.83(0.43-1.22)
Low-middle SDI	32911.41(24850.00-43611.56)	6.97(5.26-9.24)	54242.65(38164.86-72461.29)	9.35(6.58-12.50)	64.81(11.30-151.72)	34.19(-9.37-104.95)	1.36(1.15-1.57)
Middle SDI	39660.93(32221.61-46615.78)	6.87(5.58-8.08)	42712.62(31748.88-53985.24)	7.53(5.60-9.52)	7.69(-18.55-44.69)	9.66(-17.06-47.33)	0.73(0.52-0.95)
High-middle SDI	25479.35(21510.78-29833.77)	9.31(7.86-10.90)	19178.88(14878.35-23223.51)	8.31(6.44-10.06)	-24.73(-42.74, -0.60)	-10.80(-32.15-17.80)	0.23(-0.06-0.53)
High SDI	30823.93(29178.43-32348.24)	16.59(15.70-17.41)	19471.08(17170.44-21760.02)	11.29(9.95-12.61)	-36.83(-43.68, -29.75)	-31.97(-39.35, -24.35)	-1.14(-1.32, -0.96)

**Regions**							
Andean Latin America	1539.32(1085.19-2028.54)	10.36(7.31-13.66)	1185.33(825.32-1590.76)	6.55(4.56-8.79)	-23.00(-51.83-20.18)	-36.80(-60.46, -1.36)	-1.06(-1.20, -0.91)
Australasia	681.63(590.11-786.94)	14.86(12.87-17.16)	673.01(497.29-895.97)	11.74(8.68-15.63)	-1.26(-28.42-33.88)	-20.99(-42.73-7.13)	-0.60(-0.84, -0.35)
Caribbean	1282.59(939.78-1819.10)	11.24(8.23-15.94)	1843.66(1271.81-2664.23)	16.02(11.05-23.16)	43.75(1.09-103.12)	42.59(0.28-101.49)	1.29(0.88-1.70)
Central Asia	448.69(336.88-603.67)	1.80(1.35-2.42)	627.20(443.28-890.98)	2.27(1.60-3.22)	39.78(-10.36-113.38)	26.23(-19.05-92.68)	2.24(1.75-2.73)
Central Europe	2943.83(2481.05-3515.55)	9.98(8.42-11.92)	1215.51(981.35-1509.94)	6.87(5.54-8.53)	-58.71(-69.63, -43.85)	-31.23(-49.41, -6.47)	-1.12(-1.66, -0.59)
Central Latin America	6578.94(5985.40-7309.25)	10.22(9.30-11.35)	4887.59(3777.06-6352.14)	7.70(5.95-10.01)	-25.71(-43.89, -3.29)	-24.66(-43.10, -1.92)	-0.66(-1.25, -0.07)

Central Sub-Saharan Africa	628.00(254.17-1202.85)	2.48(1.00-4.75)	1007.95(678.11-1476.82)	1.72(1.16-2.52)	60.50(-11.96-270.60)	-30.80(-62.05-59.77)	-0.74(-1.10, -0.38)
East Asia	17375.53(12725.07-23038.73)	5.27(3.86-6.98)	14533.97(10424.79-19097.23)	5.44(3.90-7.14)	-16.35(-43.78-27.59)	3.19(-30.64-57.41)	1.01(0.56-1.45)
Eastern Europe	4477.83(3581.49-5824.17)	8.70(6.96-11.32)	1874.38(1430.73-2277.46)	5.29(4.04-6.43)	-58.14(-71.28, -47.00)	-39.22(-58.31, -23.04)	-1.49(-1.61, -1.38)
Eastern Sub-Saharan Africa	10064.63(6947.72-16716.11)	11.11(7.67-18.46)	21144.77(10474.68-35062.81)	11.85(5.87-19.65)	110.09(4.48-272.91)	6.64(-46.97-89.29)	0.60(0.30-0.91)
High-income Asia Pacific	6638.99(5968.00-7530.02)	18.86(16.95-21.39)	3319.78(2927.64-3744.69)	14.80(13.05-16.70)	-50.00(-58.59, -41.39)	-21.51(-35.01, -8.00)	-1.12(-1.42, -0.81)
High-income North America	12291.86(11387.24-13176.74)	19.93(18.46-21.36)	7925.34(6843.57-9067.63)	12.08(10.43-13.82)	-35.52(-44.51, -26.35)	-39.40(-47.85, -30.77)	-1.32(-1.48, -1.16)
North Africa and Middle East	8735.94(6124.25-12544.18)	6.22(4.36-8.93)	9986.29(7196.85-13356.37)	5.45(3.93-7.29)	14.31(-31.54-94.26)	-12.40(-47.53-48.87)	0.14(-0.15-0.43)

Oceania	9.93(5.26-17.31)	0.37(0.20-0.65)	15.35(8.89-26.06)	0.30(0.17-0.51)	54.60(-14.56-180.10)	-18.46(-54.94-47.74)	-1.58(-2.18, -0.98)
South Asia	31073.60(20840.55-42991.32)	7.17(4.81-9.92)	50618.87(34477.60-71561.32)	9.98(6.80-14.11)	62.90(3.07-169.37)	39.23(-11.91-130.24)	1.41(1.05-1.76)
Southeast Asia	8661.06(6086.41-12071.58)	5.07(3.56-7.07)	10278.81(7962.80-13112.13)	5.95(4.61-7.59)	18.68(-13.76-71.55)	17.37(-14.71-69.66)	0.54(0.44-0.64)
Southern Latin America	1472.04(1146.11-1935.18)	9.86(7.68-12.96)	1606.19(1148.76-2197.36)	11.08(7.92-15.16)	9.11(-26.10-62.03)	12.36(-23.91-66.85)	0.83(0.35-1.32)
Southern Sub-Saharan Africa	1017.73(757.04-1377.40)	4.92(3.66-6.66)	1498.40(1120.59-1993.48)	6.23(4.66-8.28)	47.23(-2.92-115.01)	26.57(-16.54-84.84)	1.14(0.76-1.52)
Tropical Latin America	8613.62(7390.33-9856.00)	16.07(13.78-18.38)	7497.26(5739.12-9255.19)	14.94(11.43-18.44)	-12.96(-33.48-11.78)	-7.03(-28.95-19.40)	0.22(-0.27-0.71)
Western Europe	13757.00(13112.60-14516.63)	19.37(18.46-20.44)	9151.27(7710.31-10854.85)	13.43(11.32-15.94)	-33.48(-44.40, -20.50)	-30.65(-42.03, -17.12)	-1.09(-1.29, -0.89)
Western Sub-Saharan Africa	6764.58(1913.43-10541.81)	7.70(2.18-12.00)	23295.36(5871.95-40807.72)	10.85(2.73-19.00)	244.37(96.22-398.76)	40.91(-19.71-104.09)	1.48(1.10-1.86)

Abbreviations: DALYs, disability adjusted life years; EAPC, estimated annual percentage change; SDI, Sociodemographic Index; UI, uncertainty interval.

[a] EAPC is expressed as 95% confidence interval. [b] Change shows the percentage change.

Table S3. Incidence of neuroblastoma in children, by countries and territories, 1990-2021.

	Rate per 100 000 (95%UI)				
	1990		2021		1990-2021
location	Incident cases	Incident rate	Incident cases	Incident rate	EAPC [a]
Afghanistan	0.24(0.03-1.35)	0.01(0.00-0.03)	1.78(0.54-6.62)	0.01(0.00-0.05)	3.23(2.80-3.65)
Albania	1.41(0.86-2.16)	0.13(0.08-0.19)	0.79(0.32-1.42)	0.18(0.07-0.32)	1.16(0.82-1.50)
Algeria	31.50(16.61-54.36)	0.29(0.15-0.51)	37.70(22.83-61.37)	0.28(0.17-0.46)	-0.03(-0.23-0.17)
American Samoa	0.00(0.00-0.00)	0.00(0.00-0.00)	0.00(0.00-0.00)	0.00(0.00-0.00)	-0.49(-1.40-0.43)
Andorra	0.09(0.05-0.15)	0.93(0.53-1.57)	0.03(0.02-0.05)	0.34(0.19-0.54)	-2.66(-3.05, -2.27)
Angola	3.28(0.92-8.34)	0.07(0.02-0.18)	11.12(5.92-20.27)	0.07(0.04-0.13)	0.17(0.03-0.30)
Antigua and Barbuda	0.05(0.02-0.09)	0.28(0.14-0.50)	0.07(0.05-0.12)	0.44(0.27-0.71)	1.48(1.15-1.81)
Argentina	38.87(27.04-54.82)	0.38(0.27-0.54)	46.22(31.19-66.85)	0.45(0.31-0.66)	1.16(0.83-1.49)
Armenia	0.45(0.21-0.91)	0.04(0.02-0.09)	0.63(0.32-1.09)	0.11(0.05-0.18)	5.08(4.19-5.98)

Australia	23.88(19.20-29.35)	0.63(0.51-0.78)	25.41(17.82-35.55)	0.53(0.38-0.75)	-0.48(-0.74, -0.22)
Austria	8.26(6.64-10.29)	0.61(0.49-0.76)	4.86(3.25-6.92)	0.37(0.25-0.53)	-1.30(-1.55, -1.05)
Azerbaijan	1.37(0.68-2.53)	0.06(0.03-0.10)	1.27(0.65-2.22)	0.05(0.03-0.09)	1.02(0.48-1.57)
Bahrain	0.14(0.07-0.26)	0.08(0.04-0.16)	0.60(0.34-1.02)	0.20(0.11-0.34)	5.13(4.17-6.10)
Bangladesh	70.70(33.00-135.64)	0.14(0.07-0.28)	92.18(51.65-155.24)	0.20(0.11-0.34)	1.29(0.90-1.68)
Barbados	0.60(0.39-0.88)	0.96(0.63-1.41)	0.46(0.29-0.72)	0.97(0.62-1.52)	0.47(0.20-0.74)
Belarus	8.18(5.16-12.27)	0.34(0.21-0.51)	3.34(2.03-5.02)	0.21(0.13-0.32)	-1.20(-1.73, -0.67)
Belgium	11.09(8.46-14.63)	0.61(0.47-0.81)	9.46(6.39-13.58)	0.49(0.33-0.71)	-1.12(-1.38, -0.86)
Belize	0.41(0.25-0.62)	0.50(0.30-0.76)	0.65(0.41-0.97)	0.53(0.34-0.79)	0.56(-0.41-1.54)
Benin	0.98(0.40-1.89)	0.04(0.02-0.08)	0.48(0.19-1.22)	0.01(0.00-0.02)	-6.28(-7.02, -5.53)
Bermuda	0.02(0.01-0.03)	0.14(0.08-0.23)	0.03(0.02-0.04)	0.30(0.20-0.44)	3.26(2.32-4.20)
Bhutan	0.28(0.09-0.64)	0.11(0.03-0.24)	0.40(0.16-0.78)	0.21(0.09-0.42)	2.55(2.27-2.83)
Bolivia	7.74(3.79-14.45)	0.29(0.14-0.54)	9.63(5.26-15.59)	0.28(0.15-0.45)	0.07(-0.06-0.20)

Bosnia and Herzegovina	1.24(0.67-2.18)	0.11(0.06-0.20)	0.68(0.41-1.06)	0.14(0.08-0.22)	1.83(1.26-2.40)
Botswana	0.39(0.20-0.72)	0.07(0.03-0.12)	1.24(0.56-2.65)	0.18(0.08-0.38)	4.17(3.63-4.71)
Brazil	235.69(166.68-321.40)	0.45(0.32-0.62)	236.04(165.32-314.57)	0.49(0.34-0.65)	0.68(0.11-1.25)
Brunei	0.40(0.23-0.67)	0.45(0.26-0.73)	0.40(0.25-0.60)	0.42(0.27-0.63)	0.00(-0.24-0.25)
Bulgaria	3.50(1.84-5.95)	0.20(0.11-0.34)	1.94(0.98-3.45)	0.20(0.10-0.35)	-0.40(-1.20-0.41)
Burkina Faso	1.16(0.42-2.39)	0.02(0.01-0.05)	0.49(0.16-1.34)	0.00(0.00-0.01)	-5.91(-6.60, -5.22)
Burundi	5.92(2.92-11.62)	0.23(0.11-0.44)	7.33(1.97-19.25)	0.13(0.03-0.33)	-0.76(-1.27, -0.24)
Cambodia	2.75(1.10-5.25)	0.06(0.02-0.11)	3.86(1.93-7.24)	0.08(0.04-0.14)	1.02(0.69-1.35)
Cameroon	2.36(0.93-4.77)	0.05(0.02-0.10)	1.27(0.57-3.22)	0.01(0.00-0.02)	-5.84(-6.57, -5.09)
Canada	46.21(35.35-58.34)	0.80(0.61-1.01)	31.96(21.59-49.15)	0.52(0.35-0.80)	-1.24(-1.53, -0.96)
Cape Verde	0.00(0.00-0.00)	0.00(0.00-0.00)	0.00(0.00-0.00)	0.00(0.00-0.00)	3.99(3.74-4.25)
Central African Republic	0.58(0.22-1.32)	0.05(0.02-0.11)	0.87(0.35-1.81)	0.04(0.02-0.08)	-0.45(-0.64, -0.26)
Chad	0.57(0.23-1.22)	0.02(0.01-0.04)	0.36(0.12-1.03)	0.00(0.00-0.01)	-5.86(-6.54, -5.18)

Chile	4.65(3.42-6.45)	0.12(0.09-0.16)	9.91(6.95-13.74)	0.27(0.19-0.38)	3.01(1.73-4.30)
China	486.59(308.18-743.40)	0.15(0.10-0.23)	553.02(373.13-746.77)	0.21(0.14-0.29)	2.13(1.62-2.65)
Colombia	30.62(19.95-43.78)	0.26(0.17-0.38)	37.69(23.53-56.67)	0.36(0.22-0.53)	1.52(0.78-2.26)
Comoros	0.60(0.25-1.20)	0.28(0.12-0.57)	1.07(0.46-2.24)	0.44(0.19-0.93)	1.16(0.91-1.41)
Congo	1.00(0.40-2.10)	0.09(0.04-0.20)	1.72(0.85-3.24)	0.09(0.04-0.17)	-0.01(-0.23-0.22)
Cook Islands	0.00(0.00-0.00)	0.01(0.00-0.02)	0.00(0.00-0.00)	0.02(0.00-0.06)	0.75(-0.17-1.69)
Costa Rica	4.38(3.01-6.02)	0.39(0.27-0.54)	2.61(1.75-3.75)	0.26(0.17-0.37)	-0.82(-1.07, -0.57)
Cote d'Ivoire	4.31(1.06-9.06)	0.08(0.02-0.16)	10.73(2.61-25.58)	0.09(0.02-0.22)	0.44(-0.05-0.94)
Croatia	4.45(3.29-5.94)	0.45(0.33-0.60)	3.30(2.19-4.82)	0.55(0.37-0.81)	0.86(0.55-1.17)
Cuba	10.51(7.30-15.28)	0.42(0.29-0.61)	7.58(5.21-10.90)	0.43(0.29-0.61)	1.17(0.53-1.81)
Cyprus	0.80(0.48-1.29)	0.40(0.24-0.65)	0.96(0.61-1.58)	0.44(0.28-0.72)	1.59(0.96-2.22)
Czech Republic	7.41(5.43-10.20)	0.34(0.25-0.46)	5.25(3.27-8.04)	0.31(0.19-0.47)	-0.39(-0.81-0.05)
Democratic Republic of the Congo	9.84(3.64-20.30)	0.06(0.02-0.11)	11.08(5.29-20.19)	0.03(0.01-0.05)	-1.57(-2.16, -0.98)

Denmark	2.20(1.54-3.05)	0.25(0.17-0.35)	5.53(3.55-8.17)	0.58(0.37-0.86)	2.00(1.55-2.45)
Djibouti	0.70(0.27-1.38)	0.40(0.15-0.79)	1.70(0.66-3.30)	0.41(0.16-0.80)	0.41(0.13-0.68)
Dominica	0.06(0.03-0.10)	0.23(0.12-0.41)	0.11(0.05-0.20)	0.79(0.35-1.49)	3.96(3.38-4.53)
Dominican Republic	5.35(2.71-13.05)	0.20(0.10-0.48)	16.90(9.47-26.61)	0.58(0.32-0.91)	2.20(1.29-3.12)
Ecuador	6.17(3.98-9.48)	0.16(0.10-0.25)	10.33(6.34-16.33)	0.20(0.13-0.32)	1.28(0.54-2.03)
Egypt	23.50(9.38-55.18)	0.11(0.04-0.25)	37.47(21.26-75.20)	0.10(0.06-0.20)	0.55(-0.29-1.40)
El Salvador	2.77(1.69-4.27)	0.13(0.08-0.20)	1.89(1.18-2.93)	0.10(0.06-0.16)	-0.84(-1.15, -0.53)
Equatorial Guinea	0.10(0.04-0.22)	0.05(0.02-0.11)	0.76(0.33-1.52)	0.13(0.06-0.26)	3.14(2.75-3.53)
Eritrea	2.99(1.43-5.51)	0.19(0.09-0.35)	7.91(3.08-16.96)	0.31(0.12-0.67)	1.53(1.23-1.83)
Estonia	1.19(0.74-1.87)	0.34(0.21-0.54)	0.50(0.29-0.84)	0.23(0.13-0.39)	-0.96(-1.25, -0.67)
Ethiopia	38.42(13.25-119.45)	0.16(0.05-0.49)	101.06(37.29-300.99)	0.23(0.08-0.68)	1.23(0.55-1.91)
Federated States of Micronesia	0.00(0.00-0.01)	0.01(0.00-0.01)	0.00(0.00-0.00)	0.01(0.00-0.01)	0.01(-0.21-0.23)
Fiji	0.12(0.04-0.28)	0.04(0.01-0.10)	0.12(0.04-0.28)	0.04(0.01-0.10)	-1.91(-3.17, -0.63)

Finland	1.76(1.35-2.33)	0.18(0.14-0.24)	6.08(4.28-8.76)	0.72(0.51-1.03)	4.10(3.14-5.06)
France	66.48(52.68-81.56)	0.57(0.45-0.70)	51.69(36.63-72.30)	0.45(0.32-0.62)	-0.76(-0.99, -0.52)
Gabon	0.35(0.16-0.75)	0.09(0.04-0.18)	0.75(0.39-1.31)	0.12(0.06-0.21)	1.59(1.30-1.89)
Georgia	0.14(0.07-0.25)	0.01(0.01-0.02)	0.82(0.45-1.32)	0.11(0.06-0.18)	12.66(11.11-14.22)
Germany	61.66(45.68-82.19)	0.48(0.35-0.63)	75.51(51.53-105.19)	0.63(0.43-0.88)	0.61(0.17-1.06)
Ghana	0.16(0.07-0.33)	0.00(0.00-0.00)	0.16(0.05-0.56)	0.00(0.00-0.00)	-3.39(-4.62, -2.15)
Greece	5.34(4.40-6.48)	0.26(0.22-0.32)	3.24(2.45-4.19)	0.23(0.18-0.30)	0.30(-0.04-0.64)
Greenland	0.06(0.04-0.10)	0.45(0.27-0.68)	0.01(0.00-0.02)	0.08(0.04-0.21)	-5.25(-5.66, -4.83)
Grenada	0.07(0.04-0.11)	0.20(0.11-0.33)	0.07(0.04-0.11)	0.31(0.18-0.49)	2.10(1.55-2.66)
Guam	0.00(0.00-0.00)	0.01(0.00-0.01)	0.00(0.00-0.01)	0.01(0.00-0.01)	2.94(1.43-4.47)
Guatemala	3.98(2.08-7.41)	0.10(0.05-0.18)	4.21(2.66-6.12)	0.09(0.05-0.12)	-0.61(-1.01, -0.20)
Guinea	6.26(1.50-12.43)	0.23(0.05-0.45)	11.22(2.60-25.47)	0.19(0.04-0.42)	-0.42(-0.66, -0.19)
Guinea-Bissau	0.18(0.07-0.36)	0.04(0.02-0.07)	0.04(0.01-0.11)	0.00(0.00-0.01)	-7.45(-7.86, -7.05)

Guyana	0.07(0.04-0.11)	0.02(0.01-0.04)	0.97(0.51-1.58)	0.45(0.24-0.74)	8.52(5.96-11.15)
Haiti	8.28(2.25-18.33)	0.31(0.08-0.68)	16.73(6.52-34.08)	0.38(0.15-0.78)	1.29(1.12-1.46)
Honduras	3.23(1.75-5.73)	0.15(0.08-0.26)	4.61(2.39-8.34)	0.14(0.07-0.25)	-0.17(-0.48-0.15)
Hungary	6.93(5.00-9.25)	0.33(0.23-0.43)	4.93(3.34-7.08)	0.36(0.24-0.51)	-0.26(-0.95-0.43)
Iceland	0.31(0.23-0.42)	0.50(0.37-0.66)	0.34(0.21-0.56)	0.50(0.31-0.83)	1.16(0.44-1.89)
India	496.98(269.79-795.97)	0.15(0.08-0.24)	685.49(404.16-1007.67)	0.19(0.11-0.28)	0.72(0.33-1.11)
Indonesia	61.78(29.57-114.44)	0.09(0.04-0.17)	103.30(64.34-166.82)	0.15(0.10-0.25)	1.51(1.38-1.65)
Iran	4.65(2.37-8.79)	0.02(0.01-0.03)	8.31(3.63-13.57)	0.04(0.02-0.07)	5.26(4.33-6.20)
Iraq	10.27(5.86-18.07)	0.12(0.07-0.22)	34.36(20.36-59.16)	0.26(0.15-0.44)	2.71(2.44-2.99)
Ireland	5.54(4.16-7.30)	0.56(0.42-0.74)	3.38(2.25-4.81)	0.34(0.23-0.48)	-0.77(-1.25, -0.30)
Israel	10.69(7.34-14.40)	0.70(0.48-0.94)	12.37(8.43-17.46)	0.47(0.32-0.66)	-0.83(-1.23, -0.43)
Italy	95.66(79.59-113.10)	1.04(0.86-1.23)	68.28(49.94-88.35)	0.90(0.66-1.16)	-0.61(-0.94, -0.27)
Jamaica	3.70(2.51-5.20)	0.44(0.30-0.62)	4.54(2.71-7.45)	0.78(0.46-1.28)	1.97(1.17-2.78)

Japan	178.38(152.11-206.54)	0.77(0.66-0.89)	112.45(92.43-132.17)	0.73(0.60-0.86)	-0.59(-1.03, -0.15)
Jordan	4.81(2.86-8.05)	0.29(0.18-0.49)	13.85(8.66-22.50)	0.38(0.24-0.62)	1.17(0.87-1.48)
Kazakhstan	1.19(0.69-1.94)	0.02(0.01-0.04)	2.03(1.14-3.41)	0.04(0.02-0.06)	2.46(1.99-2.92)
Kenya	10.95(5.44-21.75)	0.10(0.05-0.19)	23.68(11.49-41.29)	0.13(0.06-0.22)	1.33(1.01-1.65)
Kiribati	0.00(0.00-0.00)	0.00(0.00-0.01)	0.00(0.00-0.00)	0.00(0.00-0.01)	0.42(0.21-0.63)
Kuwait	1.60(1.07-2.22)	0.29(0.19-0.40)	3.11(2.04-4.61)	0.37(0.24-0.55)	4.51(2.32-6.76)
Kyrgyzstan	3.12(1.60-5.59)	0.19(0.10-0.33)	0.89(0.55-1.43)	0.04(0.02-0.06)	1.87(-1.21-5.04)
Laos	0.94(0.31-2.11)	0.05(0.02-0.11)	1.98(0.96-3.66)	0.09(0.04-0.16)	1.97(1.74-2.21)
Latvia	1.60(0.96-2.63)	0.28(0.17-0.46)	0.26(0.14-0.45)	0.09(0.05-0.15)	-2.01(-2.76, -1.24)
Lebanon	1.85(1.09-2.95)	0.18(0.10-0.28)	2.87(1.33-5.45)	0.22(0.10-0.43)	1.54(1.21-1.86)
Lesotho	0.28(0.14-0.52)	0.04(0.02-0.08)	0.38(0.18-0.71)	0.06(0.03-0.11)	1.72(1.26-2.18)
Liberia	0.48(0.20-0.88)	0.04(0.02-0.08)	0.09(0.03-0.23)	0.00(0.00-0.01)	-7.47(-8.55, -6.37)
Libya	6.05(3.13-11.26)	0.33(0.17-0.62)	8.82(4.72-15.90)	0.59(0.32-1.07)	2.47(1.99-2.95)

Lithuania	2.24(1.42-3.50)	0.27(0.17-0.42)	0.71(0.44-1.08)	0.17(0.11-0.27)	-0.68(-1.12, -0.24)
Luxembourg	0.42(0.32-0.53)	0.63(0.48-0.80)	0.36(0.26-0.48)	0.35(0.25-0.48)	-2.68(-3.12, -2.24)
Macedonia	1.49(0.95-2.32)	0.28(0.18-0.44)	0.53(0.29-0.85)	0.16(0.09-0.26)	-0.28(-0.75-0.19)
Madagascar	16.18(8.70-28.91)	0.30(0.16-0.53)	26.21(11.08-52.39)	0.22(0.09-0.45)	-0.60(-0.97, -0.23)
Malawi	48.62(26.23-83.74)	1.07(0.58-1.84)	73.08(25.14-163.98)	0.90(0.31-2.02)	-0.19(-0.44-0.06)
Malaysia	11.36(5.82-18.96)	0.17(0.09-0.29)	18.03(11.13-27.05)	0.24(0.15-0.36)	1.21(0.78-1.63)
Maldives	0.43(0.17-1.09)	0.41(0.16-1.03)	0.83(0.51-1.31)	0.83(0.50-1.31)	3.24(2.78-3.70)
Mali	3.74(0.74-7.89)	0.09(0.02-0.19)	8.60(1.84-20.45)	0.07(0.02-0.18)	-0.69(-0.90, -0.48)
Malta	0.98(0.67-1.34)	1.12(0.77-1.53)	0.87(0.55-1.28)	1.35(0.86-2.00)	0.65(0.23-1.08)
Marshall Islands	0.00(0.00-0.00)	0.00(0.00-0.01)	0.00(0.00-0.00)	0.01(0.00-0.01)	1.92(1.61-2.24)
Mauritania	0.40(0.17-0.80)	0.04(0.02-0.09)	0.17(0.07-0.55)	0.01(0.00-0.03)	-6.24(-6.81, -5.66)
Mauritius	0.31(0.23-0.43)	0.10(0.07-0.13)	0.42(0.30-0.59)	0.20(0.15-0.28)	2.48(1.51-3.47)
Mexico	103.09(73.75-137.47)	0.31(0.22-0.41)	81.17(55.38-116.61)	0.25(0.17-0.36)	-0.59(-1.40-0.23)

Moldova	1.58(1.05-2.36)	0.13(0.08-0.19)	0.87(0.57-1.23)	0.17(0.11-0.24)	2.17(1.74-2.60)
Mongolia	0.43(0.18-0.93)	0.05(0.02-0.10)	0.68(0.36-1.22)	0.06(0.03-0.11)	1.99(1.40-2.60)
Montenegro	0.51(0.31-0.81)	0.32(0.19-0.50)	0.17(0.10-0.28)	0.15(0.09-0.25)	-1.27(-1.62, -0.91)
Morocco	33.18(18.25-52.93)	0.34(0.19-0.54)	38.13(19.72-72.09)	0.39(0.20-0.74)	1.19(0.87-1.50)
Mozambique	17.21(8.14-35.17)	0.28(0.13-0.57)	34.34(11.12-81.27)	0.24(0.08-0.57)	-0.25(-0.53-0.04)
Myanmar	11.48(4.21-22.83)	0.08(0.03-0.15)	20.91(11.27-34.59)	0.13(0.07-0.22)	2.14(2.00-2.28)
Namibia	1.07(0.50-1.96)	0.18(0.08-0.33)	3.23(1.51-6.11)	0.39(0.18-0.74)	3.36(2.87-3.86)
Nepal	8.81(4.28-17.76)	0.10(0.05-0.21)	10.42(4.50-19.32)	0.11(0.05-0.21)	0.64(0.38-0.90)
Netherlands	26.12(20.23-33.10)	0.96(0.74-1.21)	13.84(9.61-19.72)	0.52(0.36-0.74)	-2.51(-2.87, -2.15)
New Zealand	4.46(3.30-5.96)	0.56(0.41-0.74)	3.36(2.53-4.36)	0.34(0.26-0.44)	-0.93(-1.63, -0.23)
Nicaragua	8.89(5.19-14.52)	0.49(0.28-0.80)	5.02(3.17-7.95)	0.25(0.16-0.40)	-1.53(-1.92, -1.13)
Niger	1.02(0.41-2.11)	0.03(0.01-0.05)	0.27(0.08-0.70)	0.00(0.00-0.01)	-8.81(-9.56, -8.06)
Nigeria	147.30(39.83-274.04)	0.38(0.10-0.70)	620.01(145.50-1154.47)	0.61(0.14-1.14)	2.02(1.61-2.44)

North Korea	8.95(4.71-15.20)	0.15(0.08-0.26)	4.22(1.84-8.64)	0.09(0.04-0.18)	-1.11(-1.44, -0.79)
Northern Mariana Islands	0.00(0.00-0.00)	0.00(0.00-0.00)	0.00(0.00-0.00)	0.00(0.00-0.00)	3.10(2.00-4.20)
Norway	3.01(2.46-3.59)	0.38(0.31-0.45)	1.60(1.26-2.01)	0.17(0.14-0.22)	-3.34(-3.90, -2.77)
Oman	1.18(0.35-2.51)	0.14(0.04-0.30)	1.94(1.11-3.00)	0.16(0.09-0.25)	1.15(0.43-1.89)
Pakistan	178.42(94.63-328.32)	0.36(0.19-0.67)	658.18(346.71-1128.07)	0.77(0.41-1.32)	3.48(3.02-3.94)
Palestine	2.11(1.11-4.02)	0.22(0.11-0.42)	6.37(3.76-10.07)	0.34(0.20-0.54)	2.45(1.89-3.01)
Panama	3.27(2.27-4.49)	0.39(0.27-0.54)	4.61(3.24-6.44)	0.40(0.28-0.56)	-0.37(-0.62, -0.11)
Papua New Guinea	0.05(0.02-0.12)	0.00(0.00-0.01)	0.19(0.08-0.35)	0.00(0.00-0.01)	0.80(0.51-1.09)
Paraguay	4.39(2.42-7.43)	0.26(0.15-0.45)	7.22(3.91-12.88)	0.36(0.19-0.64)	1.20(0.84-1.56)
Peru	27.42(15.03-43.30)	0.33(0.18-0.52)	18.44(10.90-29.27)	0.19(0.11-0.31)	-1.22(-1.53, -0.91)
Philippines	50.19(29.65-79.80)	0.20(0.12-0.32)	78.41(51.99-110.03)	0.23(0.15-0.32)	0.86(0.64-1.08)
Poland	24.74(19.36-31.49)	0.26(0.20-0.33)	13.33(10.04-16.84)	0.23(0.17-0.29)	-0.37(-1.50-0.77)
Portugal	21.30(16.18-27.16)	1.01(0.76-1.28)	7.53(5.39-10.78)	0.55(0.40-0.79)	-2.15(-2.36, -1.94)

Principality of Monaco	0.00(0.00-0.00)	0.01(0.01-0.02)	0.00(0.00-0.00)	0.01(0.01-0.03)	-0.99(-1.85, -0.12)
Puerto Rico	2.52(1.71-3.56)	0.25(0.17-0.36)	0.92(0.62-1.35)	0.21(0.14-0.30)	0.19(-0.51-0.90)
Qatar	0.04(0.02-0.07)	0.03(0.02-0.05)	0.30(0.15-0.52)	0.06(0.03-0.10)	3.80(2.94-4.67)
Republic of Nauru	0.00(0.00-0.00)	0.01(0.00-0.02)	0.00(0.00-0.00)	0.01(0.00-0.03)	0.75(0.48-1.02)
Republic of Niue	0.00(0.00-0.00)	0.01(0.01-0.03)	0.00(0.00-0.00)	0.07(0.04-0.15)	2.75(1.66-3.85)
Republic of Palau	0.00(0.00-0.00)	0.00(0.00-0.00)	0.00(0.00-0.00)	0.00(0.00-0.00)	-0.93(-1.24, -0.61)
Republic of San Marino	0.01(0.01-0.01)	0.20(0.13-0.32)	0.00(0.00-0.01)	0.09(0.04-0.17)	-2.35(-2.63, -2.07)
Romania	24.96(13.13-44.22)	0.45(0.24-0.79)	8.92(5.19-14.59)	0.30(0.17-0.48)	-1.06(-1.56, -0.55)
Russian Federation	95.18(65.18-133.17)	0.27(0.19-0.38)	41.99(30.40-53.58)	0.16(0.12-0.21)	-1.83(-2.01, -1.64)
Rwanda	11.27(5.82-20.33)	0.33(0.17-0.60)	16.12(6.24-32.88)	0.32(0.13-0.66)	0.15(-0.21-0.50)
Saint Kitts and Nevis	0.03(0.02-0.05)	0.21(0.12-0.35)	0.05(0.03-0.09)	0.53(0.29-0.87)	3.05(2.66-3.45)
Saint Lucia	0.13(0.08-0.21)	0.26(0.15-0.41)	0.12(0.07-0.20)	0.41(0.22-0.67)	1.49(1.09-1.89)
Saint Vincent and the Grenadines	0.01(0.00-0.01)	0.02(0.01-0.02)	0.10(0.06-0.15)	0.39(0.24-0.59)	7.39(3.48-11.45)

Samoa	0.07(0.03-0.15)	0.10(0.04-0.21)	0.11(0.03-0.26)	0.14(0.04-0.32)	1.17(0.94-1.40)
Sao Tome and Principe	0.03(0.01-0.05)	0.05(0.02-0.10)	0.01(0.00-0.01)	0.01(0.00-0.02)	-6.68(-7.69, -5.67)
Saudi Arabia	12.26(2.33-22.26)	0.19(0.04-0.34)	7.98(3.49-15.07)	0.11(0.05-0.20)	-1.48(-2.81, -0.13)
Senegal	1.65(0.60-3.33)	0.05(0.02-0.09)	0.42(0.12-1.08)	0.01(0.00-0.02)	-6.70(-7.37, -6.04)
Serbia	13.22(6.82-22.91)	0.61(0.31-1.06)	3.05(1.62-5.62)	0.23(0.12-0.42)	-3.70(-4.24, -3.16)
Seychelles	0.00(0.00-0.00)	0.00(0.00-0.00)	0.00(0.00-0.00)	0.00(0.00-0.00)	1.57(1.12-2.02)
Sierra Leone	0.80(0.33-1.55)	0.04(0.02-0.09)	0.23(0.10-0.62)	0.01(0.00-0.02)	-7.07(-7.97, -6.16)
Singapore	4.58(3.50-5.85)	0.71(0.54-0.90)	4.60(3.27-6.45)	0.57(0.40-0.79)	-0.24(-0.66-0.18)
Slovakia	3.02(1.74-4.92)	0.23(0.13-0.37)	2.50(1.33-4.51)	0.29(0.15-0.53)	0.72(0.37-1.06)
Slovenia	0.98(0.72-1.29)	0.24(0.17-0.31)	0.36(0.23-0.54)	0.12(0.07-0.17)	-1.69(-2.42, -0.94)
Solomon Islands	0.00(0.00-0.01)	0.00(0.00-0.01)	0.01(0.00-0.02)	0.00(0.00-0.01)	0.95(0.48-1.43)
Somalia	3.22(1.33-8.64)	0.08(0.03-0.22)	4.63(1.34-13.45)	0.04(0.01-0.13)	-1.41(-1.75, -1.07)
South Africa	20.40(11.73-32.69)	0.15(0.09-0.24)	26.86(17.10-40.34)	0.18(0.11-0.27)	1.01(0.46-1.56)

South Korea	61.26(37.03-94.35)	0.54(0.33-0.83)	23.56(14.54-35.10)	0.39(0.24-0.58)	-1.71(-2.16, -1.26)
South Sudan	5.94(2.35-13.92)	0.23(0.09-0.53)	8.98(3.83-17.98)	0.21(0.09-0.42)	0.38(-0.03-0.80)
Spain	54.98(43.83-67.59)	0.70(0.56-0.86)	39.33(29.83-50.55)	0.61(0.46-0.78)	-0.50(-0.79, -0.21)
Sri Lanka	9.18(5.54-14.49)	0.17(0.10-0.26)	10.42(5.81-17.38)	0.20(0.11-0.34)	0.73(0.20-1.26)
Sudan	1.24(0.21-5.96)	0.01(0.00-0.07)	5.82(2.27-19.66)	0.04(0.01-0.12)	4.10(3.74-4.47)
Suriname	0.20(0.10-0.32)	0.15(0.08-0.25)	0.36(0.20-0.58)	0.25(0.14-0.40)	2.17(1.87-2.47)
Swaziland	0.31(0.15-0.56)	0.08(0.04-0.15)	0.51(0.26-1.02)	0.12(0.06-0.25)	1.58(0.98-2.19)
Sweden	10.88(8.13-14.24)	0.70(0.53-0.92)	8.14(5.56-11.50)	0.45(0.31-0.63)	-1.95(-2.52, -1.36)
Switzerland	6.43(5.15-8.09)	0.56(0.45-0.70)	10.97(7.74-15.68)	0.82(0.58-1.18)	0.44(-0.09-0.97)
Syria	2.69(1.22-5.30)	0.05(0.02-0.09)	1.98(1.12-3.15)	0.05(0.03-0.09)	1.15(0.53-1.78)
Taiwan (Province of China)	9.09(7.15-11.16)	0.17(0.13-0.20)	6.73(5.01-8.92)	0.23(0.17-0.30)	0.88(0.28-1.47)
Tajikistan	0.05(0.03-0.10)	0.00(0.00-0.00)	0.09(0.05-0.16)	0.00(0.00-0.00)	0.49(-0.21-1.18)
Tanzania	41.69(21.38-76.51)	0.35(0.18-0.63)	119.19(50.87-238.01)	0.49(0.21-0.98)	1.70(1.32-2.08)

Thailand	38.26(23.80-59.00)	0.23(0.14-0.35)	28.44(19.40-40.67)	0.29(0.20-0.42)	0.50(0.23-0.77)
The Bahamas	0.34(0.22-0.52)	0.42(0.27-0.64)	0.33(0.20-0.55)	0.41(0.25-0.67)	-0.02(-0.37-0.33)
The Gambia	0.63(0.17-1.29)	0.14(0.04-0.28)	1.45(0.40-3.32)	0.15(0.04-0.33)	-0.06(-0.34-0.23)
Timor-Leste	0.19(0.07-0.45)	0.06(0.02-0.13)	0.35(0.18-0.63)	0.07(0.04-0.12)	0.36(0.00-0.72)
Togo	0.67(0.27-1.40)	0.04(0.02-0.08)	0.19(0.07-0.47)	0.01(0.00-0.01)	-7.09(-7.93, -6.23)
Tokelau	0.00(0.00-0.00)	0.01(0.00-0.02)	0.00(0.00-0.00)	0.10(0.02-0.28)	3.40(1.17-5.68)
Tonga	0.00(0.00-0.00)	0.00(0.00-0.01)	0.00(0.00-0.01)	0.01(0.00-0.02)	1.30(0.83-1.77)
Trinidad and Tobago	3.12(2.09-4.50)	0.77(0.52-1.11)	2.93(1.89-4.35)	1.08(0.69-1.60)	2.04(1.52-2.57)
Tunisia	15.50(9.26-24.50)	0.50(0.30-0.79)	12.18(6.93-21.35)	0.44(0.25-0.77)	0.27(-0.04-0.59)
Turkey	93.00(48.60-157.15)	0.45(0.24-0.77)	126.42(77.76-195.58)	0.68(0.42-1.06)	1.90(1.45-2.36)
Turkmenistan	0.69(0.33-1.44)	0.05(0.02-0.10)	1.07(0.59-1.85)	0.07(0.04-0.12)	2.11(1.75-2.47)
Tuvalu	0.00(0.00-0.00)	0.01(0.00-0.01)	0.00(0.00-0.00)	0.01(0.00-0.01)	0.20(-0.10-0.51)
Uganda	22.56(10.17-43.55)	0.27(0.12-0.52)	89.31(35.81-186.31)	0.45(0.18-0.94)	1.90(1.56-2.25)

Ukraine	33.14(20.48-53.17)	0.29(0.18-0.47)	20.09(12.09-33.44)	0.32(0.19-0.53)	1.17(0.49-1.84)
United Arab Emirates	0.65(0.32-1.33)	0.11(0.05-0.23)	1.77(1.03-2.75)	0.13(0.08-0.21)	1.26(0.76-1.76)
United Kingdom	127.67(106.77-148.83)	1.17(0.98-1.36)	65.30(54.27-77.28)	0.55(0.46-0.66)	-1.71(-2.28, -1.14)
United States	425.83(353.66-503.34)	0.76(0.63-0.90)	301.63(245.84-359.60)	0.51(0.41-0.61)	-1.06(-1.29, -0.83)
Uruguay	3.22(2.33-4.56)	0.39(0.28-0.56)	2.70(1.79-3.88)	0.41(0.27-0.59)	0.05(-0.39-0.48)
Uzbekistan	5.06(2.68-9.38)	0.06(0.03-0.11)	12.64(6.89-20.50)	0.13(0.07-0.20)	3.22(2.79-3.65)
Vanuatu	0.00(0.00-0.00)	0.00(0.00-0.01)	0.00(0.00-0.01)	0.00(0.00-0.01)	1.28(0.82-1.74)
Venezuela	24.10(16.53-33.37)	0.34(0.23-0.47)	19.77(12.66-29.65)	0.30(0.19-0.45)	-0.37(-0.84-0.11)
Vietnam	48.28(24.83-80.54)	0.18(0.09-0.30)	61.50(35.86-101.20)	0.25(0.14-0.41)	0.68(0.48-0.88)
Virgin Islands, U.S.	0.07(0.04-0.12)	0.23(0.13-0.38)	0.03(0.01-0.06)	0.23(0.10-0.48)	1.06(0.60-1.53)
Yemen	0.41(0.08-2.06)	0.01(0.00-0.03)	2.27(0.75-7.81)	0.02(0.01-0.06)	4.78(4.07-5.50)
Zambia	16.30(8.04-29.49)	0.43(0.21-0.79)	40.76(19.17-74.58)	0.49(0.23-0.90)	0.68(0.27-1.09)
Zimbabwe	5.54(2.56-9.68)	0.12(0.05-0.20)	10.80(5.17-18.61)	0.17(0.08-0.30)	1.43(0.91-1.96)

Abbreviations: EAPC, estimated annual percentage change; UI, uncertainty interval.

[a] EAPC is expressed as 95% confidence interval.

Table S4. Mortality of neuroblastoma in children, by countries and territories, 1990-2021.

	Rate per 100 000 (95%UI)				
	1990		2021		1990-2021
location	Number of deaths	Mortality rate	Number of deaths	Mortality rate	EAPC [a]
Afghanistan	0.11(0.02-0.59)	0.00(0.00-0.01)	0.85(0.30-3.02)	0.01(0.00-0.02)	3.26(2.83-3.69)
Albania	0.50(0.32-0.71)	0.04(0.03-0.06)	0.24(0.10-0.42)	0.05(0.02-0.10)	0.56(0.25-0.86)
Algeria	12.86(7.59-21.02)	0.12(0.07-0.20)	11.59(7.23-18.79)	0.09(0.05-0.14)	-0.57(-0.80, -0.33)
American Samoa	0.00(0.00-0.00)	0.00(0.00-0.00)	0.00(0.00-0.00)	0.00(0.00-0.00)	-0.74(-1.58-0.11)
Andorra	0.02(0.01-0.04)	0.24(0.14-0.40)	0.01(0.01-0.01)	0.09(0.05-0.14)	-2.57(-2.94, -2.20)
Angola	1.54(0.46-4.03)	0.03(0.01-0.09)	4.57(2.62-8.05)	0.03(0.02-0.05)	0.02(-0.16-0.20)
Antigua and Barbuda	0.02(0.01-0.03)	0.10(0.05-0.17)	0.02(0.01-0.03)	0.13(0.09-0.21)	1.20(0.77-1.64)
Argentina	14.01(10.64-18.70)	0.14(0.10-0.18)	14.51(10.15-20.23)	0.14(0.10-0.20)	0.59(0.18-1.00)
Armenia	0.17(0.08-0.32)	0.02(0.01-0.03)	0.20(0.10-0.34)	0.03(0.02-0.06)	4.29(3.60-4.99)

Australia	6.30(5.31-7.48)	0.17(0.14-0.20)	6.75(4.92-9.22)	0.14(0.10-0.19)	-0.49(-0.75, -0.23)
Austria	2.53(2.18-2.98)	0.19(0.16-0.22)	1.29(0.89-1.78)	0.10(0.07-0.14)	-1.65(-1.83, -1.46)
Azerbaijan	0.56(0.30-0.96)	0.02(0.01-0.04)	0.46(0.26-0.77)	0.02(0.01-0.03)	0.47(-0.00-0.94)
Bahrain	0.05(0.03-0.09)	0.03(0.02-0.05)	0.19(0.11-0.30)	0.06(0.04-0.10)	4.45(3.62-5.29)
Bangladesh	33.29(18.08-53.93)	0.07(0.04-0.11)	33.50(20.18-52.05)	0.07(0.04-0.11)	0.25(-0.02-0.52)
Barbados	0.21(0.15-0.29)	0.34(0.25-0.46)	0.14(0.09-0.21)	0.30(0.20-0.45)	-0.13(-0.33-0.07)
Belarus	2.92(1.97-4.19)	0.12(0.08-0.17)	1.07(0.70-1.66)	0.07(0.04-0.11)	-1.42(-1.85, -1.00)
Belgium	3.41(2.68-4.27)	0.19(0.15-0.24)	2.52(1.75-3.48)	0.13(0.09-0.18)	-1.48(-1.66, -1.29)
Belize	0.16(0.12-0.23)	0.20(0.15-0.28)	0.23(0.16-0.32)	0.19(0.13-0.26)	0.08(-0.81-0.98)
Benin	0.46(0.21-0.83)	0.02(0.01-0.03)	0.20(0.09-0.48)	0.00(0.00-0.01)	-6.42(-7.07, -5.75)
Bermuda	0.01(0.00-0.01)	0.05(0.03-0.08)	0.01(0.00-0.01)	0.08(0.05-0.11)	2.34(1.37-3.32)
Bhutan	0.13(0.05-0.28)	0.05(0.02-0.11)	0.14(0.06-0.26)	0.08(0.03-0.14)	1.61(1.34-1.87)
Bolivia	3.66(2.11-5.99)	0.14(0.08-0.22)	3.98(2.48-6.02)	0.11(0.07-0.17)	-0.58(-0.70, -0.46)

Bosnia and Herzegovina	0.45(0.27-0.75)	0.04(0.02-0.07)	0.22(0.14-0.33)	0.04(0.03-0.07)	1.26(0.83-1.70)
Botswana	0.18(0.11-0.31)	0.03(0.02-0.05)	0.51(0.26-1.02)	0.07(0.04-0.15)	3.47(3.06-3.88)
Brazil	96.46(82.56-110.09)	0.19(0.16-0.21)	83.60(63.77-103.45)	0.17(0.13-0.21)	0.24(-0.26-0.74)
Brunei	0.14(0.09-0.24)	0.16(0.10-0.26)	0.12(0.08-0.18)	0.13(0.09-0.19)	-0.17(-0.51-0.16)
Bulgaria	1.26(0.69-2.05)	0.07(0.04-0.12)	0.61(0.32-1.05)	0.06(0.03-0.11)	-1.05(-1.79, -0.31)
Burkina Faso	0.54(0.22-1.05)	0.01(0.00-0.02)	0.23(0.10-0.59)	0.00(0.00-0.01)	-5.83(-6.52, -5.14)
Burundi	2.76(1.58-5.00)	0.11(0.06-0.19)	3.46(1.08-8.58)	0.06(0.02-0.15)	-0.74(-1.25, -0.23)
Cambodia	1.30(0.65-2.15)	0.03(0.01-0.05)	1.59(0.91-2.68)	0.03(0.02-0.05)	0.38(0.17-0.60)
Cameroon	1.11(0.50-2.02)	0.02(0.01-0.04)	0.54(0.27-1.50)	0.00(0.00-0.01)	-6.13(-6.74, -5.51)
Canada	12.29(9.94-15.22)	0.21(0.17-0.26)	8.64(5.95-13.32)	0.14(0.10-0.22)	-1.22(-1.50, -0.94)
Cape Verde	0.00(0.00-0.00)	0.00(0.00-0.00)	0.00(0.00-0.00)	0.00(0.00-0.00)	3.39(3.12-3.65)
Central African Republic	0.27(0.11-0.55)	0.02(0.01-0.04)	0.41(0.19-0.79)	0.02(0.01-0.03)	-0.44(-0.63, -0.26)
Chad	0.27(0.12-0.51)	0.01(0.00-0.02)	0.17(0.07-0.50)	0.00(0.00-0.01)	-5.78(-6.45, -5.10)

Chile	1.67(1.35-2.06)	0.04(0.03-0.05)	3.17(2.36-4.21)	0.09(0.06-0.12)	2.52(1.37-3.68)
China	189.99(137.75-253.36)	0.06(0.04-0.08)	162.72(116.13-213.02)	0.06(0.04-0.08)	1.07(0.62-1.51)
Colombia	12.58(9.60-15.99)	0.11(0.08-0.14)	11.81(7.99-16.57)	0.11(0.08-0.16)	0.72(0.01-1.44)
Comoros	0.28(0.14-0.50)	0.13(0.07-0.24)	0.43(0.21-0.81)	0.18(0.09-0.34)	0.87(0.60-1.14)
Congo	0.47(0.20-0.90)	0.04(0.02-0.09)	0.71(0.40-1.21)	0.04(0.02-0.06)	-0.44(-0.65, -0.23)
Cook Islands	0.00(0.00-0.00)	0.00(0.00-0.01)	0.00(0.00-0.00)	0.01(0.00-0.02)	0.08(-0.87-1.04)
Costa Rica	1.55(1.17-2.01)	0.14(0.10-0.18)	0.83(0.59-1.15)	0.08(0.06-0.11)	-1.38(-1.60, -1.15)
Cote d'Ivoire	2.01(0.45-3.80)	0.04(0.01-0.07)	4.37(1.10-9.24)	0.04(0.01-0.08)	0.06(-0.31-0.43)
Croatia	1.38(1.06-1.74)	0.14(0.11-0.18)	0.88(0.60-1.24)	0.15(0.10-0.21)	0.14(-0.15-0.44)
Cuba	3.74(2.80-5.01)	0.15(0.11-0.20)	2.33(1.66-3.20)	0.13(0.09-0.18)	0.74(0.20-1.29)
Cyprus	0.24(0.16-0.39)	0.12(0.08-0.20)	0.26(0.17-0.40)	0.12(0.08-0.18)	0.90(0.38-1.42)
Czech Republic	2.32(1.78-3.09)	0.11(0.08-0.14)	1.43(0.92-2.18)	0.08(0.05-0.13)	-1.10(-1.54, -0.66)
Democratic Republic of the Congo	4.62(1.86-8.96)	0.03(0.01-0.05)	5.27(3.05-8.75)	0.01(0.01-0.02)	-1.53(-2.12, -0.94)

Denmark	0.68(0.50-0.89)	0.08(0.06-0.10)	1.47(0.99-2.14)	0.15(0.10-0.22)	1.35(0.85-1.85)
Djibouti	0.32(0.15-0.57)	0.19(0.08-0.33)	0.69(0.29-1.30)	0.17(0.07-0.32)	0.00(-0.31-0.32)
Dominica	0.02(0.01-0.04)	0.09(0.06-0.15)	0.04(0.02-0.07)	0.28(0.14-0.53)	3.51(3.11-3.91)
Dominican Republic	2.15(1.26-4.88)	0.08(0.05-0.18)	5.95(3.60-9.25)	0.20(0.12-0.31)	1.64(0.83-2.46)
Ecuador	2.58(1.91-3.47)	0.07(0.05-0.09)	3.80(2.59-5.71)	0.08(0.05-0.11)	0.89(0.30-1.49)
Egypt	9.62(4.12-22.32)	0.04(0.02-0.10)	13.40(8.06-26.62)	0.04(0.02-0.07)	-0.09(-0.80-0.63)
El Salvador	1.14(0.77-1.61)	0.05(0.04-0.07)	0.69(0.47-0.99)	0.04(0.03-0.05)	-1.22(-1.44, -1.01)
Equatorial Guinea	0.05(0.02-0.10)	0.02(0.01-0.05)	0.28(0.13-0.52)	0.05(0.02-0.09)	2.15(1.76-2.53)
Eritrea	1.40(0.78-2.32)	0.09(0.05-0.15)	3.70(1.65-7.04)	0.15(0.07-0.28)	1.55(1.25-1.86)
Estonia	0.42(0.28-0.65)	0.12(0.08-0.19)	0.14(0.08-0.23)	0.06(0.04-0.11)	-1.47(-1.78, -1.17)
Ethiopia	17.90(7.29-59.22)	0.07(0.03-0.24)	42.77(18.05-120.06)	0.10(0.04-0.27)	0.94(0.35-1.53)
Federated States of Micronesia	0.00(0.00-0.00)	0.00(0.00-0.01)	0.00(0.00-0.00)	0.00(0.00-0.00)	-0.65(-0.83, -0.48)
Fiji	0.05(0.02-0.11)	0.02(0.01-0.04)	0.05(0.02-0.11)	0.02(0.01-0.04)	-1.92(-3.19, -0.63)

Finland	0.55(0.43-0.70)	0.06(0.04-0.07)	1.64(1.19-2.27)	0.19(0.14-0.27)	3.58(2.76-4.41)
France	20.41(17.52-23.23)	0.17(0.15-0.20)	13.75(10.10-18.86)	0.12(0.09-0.16)	-1.23(-1.45, -1.01)
Gabon	0.17(0.09-0.33)	0.04(0.02-0.08)	0.31(0.18-0.51)	0.05(0.03-0.08)	0.95(0.72-1.17)
Georgia	0.05(0.03-0.08)	0.00(0.00-0.01)	0.30(0.18-0.47)	0.04(0.02-0.06)	12.57(11.03-14.13)
Germany	19.05(15.16-23.99)	0.15(0.12-0.19)	20.02(14.65-27.00)	0.17(0.12-0.23)	0.22(-0.10-0.54)
Ghana	0.08(0.04-0.14)	0.00(0.00-0.00)	0.07(0.02-0.22)	0.00(0.00-0.00)	-3.98(-5.17, -2.77)
Global	1642.64(1373.57-1956.38)	0.09(0.08-0.11)	1976.92(1445.04-2528.54)	0.10(0.07-0.13)	0.44(0.32-0.57)
Greece	1.43(1.24-1.63)	0.07(0.06-0.08)	0.87(0.68-1.08)	0.06(0.05-0.08)	0.26(-0.07-0.59)
Greenland	0.02(0.01-0.03)	0.16(0.10-0.23)	0.00(0.00-0.01)	0.03(0.01-0.06)	-5.84(-6.20, -5.48)
Grenada	0.03(0.02-0.04)	0.08(0.05-0.13)	0.02(0.02-0.04)	0.11(0.07-0.17)	1.64(1.21-2.07)
Guam	0.00(0.00-0.00)	0.00(0.00-0.00)	0.00(0.00-0.00)	0.00(0.00-0.00)	2.83(1.47-4.21)
Guatemala	1.88(1.15-3.13)	0.05(0.03-0.08)	1.51(1.04-2.14)	0.03(0.02-0.04)	-1.48(-1.74, -1.21)
Guinea	2.91(0.76-5.14)	0.11(0.03-0.19)	5.26(1.27-11.40)	0.09(0.02-0.19)	-0.39(-0.62, -0.15)

Guinea-Bissau	0.09(0.04-0.15)	0.02(0.01-0.03)	0.02(0.01-0.05)	0.00(0.00-0.01)	-7.32(-7.72, -6.91)
Guyana	0.03(0.02-0.05)	0.01(0.01-0.02)	0.40(0.25-0.61)	0.19(0.12-0.29)	7.98(5.53-10.48)
Haiti	3.88(1.17-7.88)	0.14(0.04-0.29)	7.84(3.27-14.73)	0.18(0.08-0.34)	1.29(1.12-1.46)
Honduras	1.54(0.96-2.38)	0.07(0.04-0.11)	1.92(1.10-3.15)	0.06(0.03-0.10)	-0.67(-0.85, -0.49)
Hungary	2.50(1.99-3.08)	0.12(0.09-0.14)	1.31(0.89-1.87)	0.09(0.06-0.13)	-0.59(-1.26-0.09)
Iceland	0.08(0.07-0.11)	0.13(0.10-0.17)	0.10(0.06-0.15)	0.14(0.09-0.23)	1.17(0.48-1.87)
India	228.45(140.24-315.75)	0.07(0.04-0.10)	263.49(176.86-360.16)	0.07(0.05-0.10)	0.04(-0.30-0.39)
Indonesia	28.37(15.96-48.98)	0.04(0.02-0.07)	38.86(26.71-59.10)	0.06(0.04-0.09)	0.91(0.78-1.04)
Iran	1.67(0.93-3.00)	0.01(0.00-0.01)	2.67(1.13-4.27)	0.01(0.01-0.02)	4.83(3.89-5.77)
Iraq	4.21(2.60-6.62)	0.05(0.03-0.08)	10.55(6.44-17.00)	0.08(0.05-0.13)	1.88(1.65-2.12)
Ireland	1.72(1.41-2.13)	0.17(0.14-0.22)	0.90(0.63-1.25)	0.09(0.06-0.13)	-1.36(-1.68, -1.04)
Israel	3.26(2.38-4.22)	0.21(0.16-0.28)	3.28(2.34-4.50)	0.12(0.09-0.17)	-1.50(-1.87, -1.13)
Italy	27.96(25.33-30.56)	0.30(0.27-0.33)	18.40(13.95-23.17)	0.24(0.18-0.30)	-0.77(-1.05, -0.49)

Jamaica	1.31(0.99-1.75)	0.16(0.12-0.21)	1.62(0.97-2.53)	0.28(0.17-0.43)	2.00(1.20-2.81)
Japan	52.35(49.87-54.77)	0.23(0.22-0.24)	30.27(26.80-33.73)	0.20(0.17-0.22)	-0.73(-1.09, -0.37)
Jordan	1.71(1.11-2.62)	0.10(0.07-0.16)	4.29(2.79-6.54)	0.12(0.08-0.18)	0.50(0.24-0.76)
Kazakhstan	0.43(0.26-0.68)	0.01(0.01-0.01)	0.64(0.37-1.03)	0.01(0.01-0.02)	1.79(1.53-2.04)
Kenya	4.77(2.86-8.44)	0.04(0.03-0.08)	9.75(5.37-15.69)	0.05(0.03-0.08)	1.20(0.91-1.50)
Kiribati	0.00(0.00-0.00)	0.00(0.00-0.00)	0.00(0.00-0.00)	0.00(0.00-0.00)	0.43(0.22-0.65)
Kuwait	0.49(0.36-0.67)	0.09(0.06-0.12)	0.83(0.54-1.19)	0.10(0.06-0.14)	3.99(1.87-6.16)
Kyrgyzstan	1.26(0.70-2.13)	0.08(0.04-0.13)	0.32(0.22-0.47)	0.01(0.01-0.02)	1.84(-1.23-5.02)
Laos	0.45(0.17-0.89)	0.02(0.01-0.05)	0.82(0.46-1.39)	0.04(0.02-0.06)	1.53(1.39-1.66)
Latvia	0.57(0.38-0.89)	0.10(0.07-0.16)	0.08(0.05-0.14)	0.03(0.02-0.05)	-2.14(-2.93, -1.35)
Lebanon	0.65(0.41-0.98)	0.06(0.04-0.09)	0.88(0.44-1.54)	0.07(0.03-0.12)	0.89(0.65-1.14)
Lesotho	0.13(0.08-0.22)	0.02(0.01-0.03)	0.18(0.10-0.31)	0.03(0.02-0.05)	1.78(1.33-2.23)
Liberia	0.23(0.11-0.38)	0.02(0.01-0.03)	0.04(0.01-0.09)	0.00(0.00-0.00)	-7.94(-8.94, -6.92)

Libya	2.13(1.21-3.86)	0.12(0.07-0.21)	2.72(1.48-4.61)	0.18(0.10-0.31)	1.83(1.37-2.29)
Lithuania	0.69(0.45-1.03)	0.08(0.05-0.12)	0.22(0.14-0.33)	0.05(0.03-0.08)	-0.61(-1.04, -0.17)
Luxembourg	0.13(0.11-0.15)	0.19(0.16-0.23)	0.09(0.07-0.12)	0.09(0.07-0.12)	-3.13(-3.53, -2.74)
Macedonia	0.53(0.37-0.75)	0.10(0.07-0.14)	0.17(0.10-0.27)	0.05(0.03-0.08)	-0.81(-1.27, -0.35)
Madagascar	7.54(4.68-11.86)	0.14(0.09-0.22)	12.33(5.92-21.98)	0.11(0.05-0.19)	-0.58(-0.96, -0.21)
Malawi	22.61(14.62-33.99)	0.50(0.32-0.75)	34.11(12.91-71.01)	0.42(0.16-0.87)	-0.26(-0.49, -0.03)
Malaysia	4.73(2.65-7.16)	0.07(0.04-0.11)	6.56(4.35-9.19)	0.09(0.06-0.12)	0.81(0.34-1.28)
Maldives	0.18(0.07-0.44)	0.17(0.07-0.42)	0.25(0.16-0.39)	0.25(0.16-0.39)	2.01(1.64-2.38)
Mali	1.74(0.36-3.27)	0.04(0.01-0.08)	4.03(0.91-9.06)	0.03(0.01-0.08)	-0.65(-0.87, -0.44)
Malta	0.30(0.22-0.41)	0.34(0.25-0.46)	0.24(0.15-0.35)	0.37(0.24-0.55)	0.16(-0.28-0.60)
Marshall Islands	0.00(0.00-0.00)	0.00(0.00-0.00)	0.00(0.00-0.00)	0.00(0.00-0.01)	1.50(1.09-1.91)
Mauritania	0.19(0.09-0.34)	0.02(0.01-0.04)	0.06(0.03-0.19)	0.00(0.00-0.01)	-6.94(-7.52, -6.37)
Mauritius	0.11(0.09-0.14)	0.03(0.03-0.04)	0.15(0.11-0.19)	0.07(0.06-0.09)	2.50(1.54-3.48)

Mexico	41.38(37.14-46.74)	0.12(0.11-0.14)	28.87(22.15-38.66)	0.09(0.07-0.12)	-0.93(-1.65, -0.21)
Moldova	0.56(0.41-0.84)	0.05(0.03-0.07)	0.27(0.19-0.36)	0.05(0.04-0.07)	1.61(1.15-2.08)
Mongolia	0.21(0.09-0.41)	0.02(0.01-0.05)	0.25(0.14-0.43)	0.02(0.01-0.04)	0.79(0.32-1.26)
Montenegro	0.16(0.10-0.24)	0.10(0.06-0.15)	0.05(0.03-0.08)	0.04(0.03-0.07)	-1.43(-1.83, -1.02)
Morocco	13.54(8.27-20.25)	0.14(0.08-0.21)	13.60(7.64-23.91)	0.14(0.08-0.24)	0.54(0.29-0.79)
Mozambique	7.98(4.46-15.03)	0.13(0.07-0.24)	16.13(5.97-36.71)	0.11(0.04-0.26)	-0.21(-0.49-0.07)
Myanmar	5.45(2.23-10.28)	0.04(0.02-0.07)	8.59(5.27-13.49)	0.05(0.03-0.09)	1.48(1.39-1.57)
Namibia	0.50(0.25-0.79)	0.08(0.04-0.13)	1.32(0.67-2.17)	0.16(0.08-0.26)	2.66(2.28-3.03)
Nepal	4.13(2.34-7.47)	0.05(0.03-0.09)	4.31(2.02-7.61)	0.05(0.02-0.08)	0.03(-0.11-0.17)
Netherlands	6.93(5.67-8.62)	0.25(0.21-0.32)	3.72(2.62-5.16)	0.14(0.10-0.19)	-2.46(-2.81, -2.09)
New Zealand	1.42(1.14-1.78)	0.18(0.14-0.22)	0.94(0.73-1.19)	0.10(0.07-0.12)	-1.17(-1.83, -0.51)
Nicaragua	3.62(2.35-5.48)	0.20(0.13-0.30)	1.80(1.17-2.71)	0.09(0.06-0.14)	-2.09(-2.40, -1.77)
Niger	0.48(0.21-0.99)	0.01(0.01-0.02)	0.13(0.05-0.33)	0.00(0.00-0.00)	-8.71(-9.46, -7.97)

Nigeria	64.63(17.68-103.03)	0.17(0.05-0.26)	248.66(57.27-441.58)	0.24(0.06-0.43)	1.69(1.30-2.07)
North Korea	3.66(2.17-6.00)	0.06(0.04-0.10)	1.52(0.68-3.02)	0.03(0.01-0.06)	-1.69(-1.96, -1.42)
Northern Mariana Islands	0.00(0.00-0.00)	0.00(0.00-0.00)	0.00(0.00-0.00)	0.00(0.00-0.00)	3.08(1.99-4.18)
Norway	0.95(0.86-1.02)	0.12(0.11-0.13)	0.48(0.39-0.59)	0.05(0.04-0.06)	-3.41(-3.86, -2.96)
Oman	0.42(0.12-0.86)	0.05(0.01-0.10)	0.60(0.36-0.87)	0.05(0.03-0.07)	0.53(-0.12-1.19)
Pakistan	83.42(52.23-132.13)	0.17(0.11-0.27)	268.53(165.09-426.35)	0.31(0.19-0.50)	2.97(2.58-3.37)
Palestine	0.74(0.42-1.36)	0.08(0.04-0.14)	1.96(1.20-2.96)	0.11(0.06-0.16)	2.24(1.67-2.81)
Panama	1.16(0.90-1.46)	0.14(0.11-0.17)	1.42(1.06-1.89)	0.12(0.09-0.16)	-0.77(-1.01, -0.52)
Papua New Guinea	0.03(0.01-0.05)	0.00(0.00-0.00)	0.07(0.03-0.14)	0.00(0.00-0.00)	0.58(0.30-0.86)
Paraguay	1.79(1.08-2.74)	0.11(0.06-0.16)	2.56(1.51-4.24)	0.13(0.08-0.21)	0.98(0.68-1.28)
Peru	11.25(7.15-15.83)	0.14(0.09-0.19)	5.83(3.71-8.89)	0.06(0.04-0.09)	-2.04(-2.23, -1.85)
Philippines	20.66(13.98-30.23)	0.08(0.06-0.12)	29.25(21.37-37.43)	0.09(0.06-0.11)	0.67(0.48-0.86)
Poland	8.90(8.12-9.76)	0.09(0.08-0.10)	4.15(3.38-5.01)	0.07(0.06-0.09)	-0.78(-1.80-0.25)

Portugal	6.60(5.35-7.94)	0.31(0.25-0.38)	2.00(1.46-2.80)	0.15(0.11-0.21)	-2.85(-3.11, -2.60)
Principality of Monaco	0.00(0.00-0.00)	0.00(0.00-0.00)	0.00(0.00-0.00)	0.00(0.00-0.01)	-0.94(-1.78, -0.11)
Puerto Rico	0.89(0.68-1.20)	0.09(0.07-0.12)	0.28(0.20-0.40)	0.06(0.05-0.09)	-0.18(-0.78-0.42)
Qatar	0.01(0.01-0.02)	0.01(0.01-0.02)	0.08(0.04-0.13)	0.02(0.01-0.03)	2.91(2.01-3.81)
Republic of Nauru	0.00(0.00-0.00)	0.00(0.00-0.01)	0.00(0.00-0.00)	0.00(0.00-0.01)	0.23(0.09-0.38)
Republic of Niue	0.00(0.00-0.00)	0.01(0.00-0.01)	0.00(0.00-0.00)	0.03(0.01-0.05)	2.17(1.08-3.28)
Republic of Palau	0.00(0.00-0.00)	0.00(0.00-0.00)	0.00(0.00-0.00)	0.00(0.00-0.00)	-1.08(-1.33, -0.84)
Republic of San Marino	0.00(0.00-0.00)	0.05(0.03-0.08)	0.00(0.00-0.00)	0.02(0.01-0.05)	-2.29(-2.56, -2.02)
Romania	8.92(5.14-15.06)	0.16(0.09-0.27)	2.79(1.68-4.50)	0.09(0.06-0.15)	-1.66(-2.04, -1.28)
Russian Federation	33.87(26.41-44.12)	0.10(0.08-0.13)	13.25(10.45-15.54)	0.05(0.04-0.06)	-2.44(-2.71, -2.17)
Rwanda	5.24(3.08-8.58)	0.15(0.09-0.25)	6.54(2.81-11.98)	0.13(0.06-0.24)	-0.20(-0.50-0.11)
Saint Kitts and Nevis	0.01(0.01-0.02)	0.09(0.06-0.13)	0.02(0.01-0.03)	0.19(0.11-0.30)	2.48(2.21-2.75)
Saint Lucia	0.05(0.04-0.08)	0.11(0.07-0.16)	0.04(0.03-0.07)	0.15(0.09-0.24)	1.29(1.03-1.55)

Saint Vincent and the Grenadines	0.00(0.00-0.00)	0.01(0.00-0.01)	0.04(0.02-0.05)	0.14(0.10-0.21)	6.89(3.09-10.83)
Samoa	0.03(0.01-0.06)	0.04(0.02-0.08)	0.04(0.01-0.08)	0.05(0.02-0.10)	0.49(0.35-0.63)
Sao Tome and Principe	0.01(0.01-0.02)	0.02(0.01-0.04)	0.00(0.00-0.01)	0.00(0.00-0.01)	-7.29(-8.33, -6.24)
Saudi Arabia	5.02(0.95-8.84)	0.08(0.01-0.13)	2.52(1.04-4.81)	0.03(0.01-0.06)	-2.28(-3.56, -0.98)
Senegal	0.77(0.33-1.40)	0.02(0.01-0.04)	0.18(0.06-0.41)	0.00(0.00-0.01)	-7.16(-7.79, -6.53)
Serbia	4.73(2.66-7.73)	0.22(0.12-0.36)	0.97(0.53-1.77)	0.07(0.04-0.13)	-4.10(-4.59, -3.60)
Seychelles	0.00(0.00-0.00)	0.00(0.00-0.00)	0.00(0.00-0.00)	0.00(0.00-0.00)	1.37(0.96-1.78)
Sierra Leone	0.37(0.17-0.65)	0.02(0.01-0.04)	0.10(0.04-0.25)	0.00(0.00-0.01)	-7.15(-7.97, -6.32)
Singapore	1.42(1.15-1.71)	0.22(0.18-0.26)	1.24(0.90-1.67)	0.15(0.11-0.21)	-0.93(-1.38, -0.48)
Slovakia	1.09(0.68-1.69)	0.08(0.05-0.13)	0.77(0.42-1.39)	0.09(0.05-0.16)	0.60(0.29-0.91)
Slovenia	0.30(0.24-0.38)	0.07(0.06-0.09)	0.10(0.06-0.14)	0.03(0.02-0.05)	-2.37(-3.04, -1.68)
Solomon Islands	0.00(0.00-0.00)	0.00(0.00-0.00)	0.00(0.00-0.01)	0.00(0.00-0.00)	0.79(0.31-1.28)
Somalia	1.50(0.70-3.73)	0.04(0.02-0.10)	2.16(0.73-6.39)	0.02(0.01-0.06)	-1.40(-1.74, -1.07)

South Africa	8.30(5.39-11.99)	0.06(0.04-0.09)	9.71(7.10-13.94)	0.06(0.05-0.09)	0.55(0.11-0.99)
South Korea	22.15(14.42-32.42)	0.19(0.13-0.29)	6.40(4.06-9.45)	0.11(0.07-0.16)	-2.67(-3.11, -2.23)
South Sudan	2.76(1.24-5.68)	0.11(0.05-0.22)	4.19(2.17-7.63)	0.10(0.05-0.18)	0.40(-0.02-0.82)
Spain	17.15(14.84-19.73)	0.22(0.19-0.25)	10.57(8.24-13.50)	0.16(0.13-0.21)	-0.83(-1.02, -0.64)
Sri Lanka	3.83(2.60-5.59)	0.07(0.05-0.10)	3.28(1.94-5.38)	0.06(0.04-0.11)	0.02(-0.54-0.58)
Sudan	0.58(0.11-2.63)	0.01(0.00-0.03)	2.05(0.84-6.68)	0.01(0.01-0.04)	3.11(2.70-3.53)
Suriname	0.08(0.05-0.12)	0.06(0.04-0.09)	0.13(0.07-0.20)	0.09(0.05-0.14)	1.67(1.33-2.01)
Swaziland	0.14(0.08-0.25)	0.04(0.02-0.06)	0.21(0.12-0.38)	0.05(0.03-0.09)	1.18(0.71-1.66)
Sweden	2.94(2.31-3.70)	0.19(0.15-0.24)	2.20(1.53-3.09)	0.12(0.08-0.17)	-1.90(-2.49, -1.32)
Switzerland	1.70(1.39-2.08)	0.15(0.12-0.18)	2.93(2.10-4.08)	0.22(0.16-0.31)	0.45(-0.08-0.98)
Syria	1.11(0.58-2.15)	0.02(0.01-0.04)	0.63(0.37-0.96)	0.02(0.01-0.03)	0.42(-0.07-0.91)
Taiwan (Province of China)	2.85(2.46-3.29)	0.05(0.04-0.06)	1.83(1.43-2.40)	0.06(0.05-0.08)	0.22(-0.28-0.72)
Tajikistan	0.02(0.01-0.04)	0.00(0.00-0.00)	0.03(0.02-0.05)	0.00(0.00-0.00)	0.07(-0.50-0.64)

Tanzania	19.39(11.94-32.36)	0.16(0.10-0.27)	48.17(22.21-85.27)	0.20(0.09-0.35)	1.29(0.99-1.60)
Thailand	13.75(9.17-19.36)	0.08(0.05-0.11)	8.85(6.37-12.17)	0.09(0.07-0.12)	-0.16(-0.50-0.17)
The Bahamas	0.12(0.08-0.17)	0.15(0.10-0.21)	0.12(0.08-0.19)	0.15(0.10-0.23)	0.01(-0.33-0.36)
The Gambia	0.30(0.08-0.55)	0.06(0.02-0.12)	0.59(0.18-1.26)	0.06(0.02-0.13)	-0.63(-0.97, -0.28)
Timor-Leste	0.09(0.04-0.19)	0.03(0.01-0.06)	0.15(0.09-0.24)	0.03(0.02-0.05)	-0.26(-0.59-0.06)
Togo	0.31(0.14-0.61)	0.02(0.01-0.03)	0.08(0.03-0.21)	0.00(0.00-0.01)	-7.36(-8.08, -6.63)
Tokelau	0.00(0.00-0.00)	0.00(0.00-0.01)	0.00(0.00-0.00)	0.03(0.01-0.09)	2.77(0.55-5.05)
Tonga	0.00(0.00-0.00)	0.00(0.00-0.00)	0.00(0.00-0.00)	0.00(0.00-0.01)	0.64(0.24-1.05)
Trinidad and Tobago	1.28(1.01-1.61)	0.31(0.25-0.40)	1.04(0.73-1.43)	0.38(0.27-0.53)	1.51(1.13-1.90)
Tunisia	5.47(3.42-8.65)	0.18(0.11-0.28)	3.77(2.20-6.55)	0.14(0.08-0.24)	-0.38(-0.59, -0.17)
Turkey	38.10(22.18-60.98)	0.19(0.11-0.30)	39.14(24.64-57.91)	0.21(0.13-0.31)	0.94(0.61-1.28)
Turkmenistan	0.29(0.16-0.54)	0.02(0.01-0.04)	0.39(0.24-0.64)	0.03(0.02-0.04)	1.47(1.13-1.82)
Tuvalu	0.00(0.00-0.00)	0.00(0.00-0.01)	0.00(0.00-0.00)	0.00(0.00-0.01)	-0.38(-0.60, -0.15)

Uganda	10.46(5.50-18.08)	0.12(0.07-0.21)	36.15(15.80-70.77)	0.18(0.08-0.36)	1.56(1.27-1.84)
Ukraine	11.73(7.91-17.81)	0.10(0.07-0.16)	6.29(3.84-9.68)	0.10(0.06-0.15)	0.74(0.23-1.25)
United Arab Emirates	0.27(0.15-0.49)	0.05(0.03-0.08)	0.55(0.35-0.83)	0.04(0.03-0.06)	0.78(0.20-1.37)
United Kingdom	37.88(36.16-39.83)	0.35(0.33-0.36)	17.39(15.19-19.37)	0.15(0.13-0.16)	-2.13(-2.65, -1.61)
United States	127.70(117.80-137.34)	0.23(0.21-0.25)	82.38(70.44-94.39)	0.14(0.12-0.16)	-1.32(-1.50, -1.13)
Uruguay	1.17(0.89-1.56)	0.14(0.11-0.19)	0.86(0.60-1.21)	0.13(0.09-0.18)	-0.45(-0.78, -0.12)
Uzbekistan	2.10(1.20-3.61)	0.02(0.01-0.04)	4.57(2.71-7.17)	0.05(0.03-0.07)	2.78(2.46-3.10)
Vanuatu	0.00(0.00-0.00)	0.00(0.00-0.00)	0.00(0.00-0.00)	0.00(0.00-0.00)	1.03(0.56-1.51)
Venezuela	9.94(8.27-12.05)	0.14(0.12-0.17)	7.21(5.06-10.11)	0.11(0.08-0.15)	-0.46(-0.86, -0.07)
Vietnam	19.63(10.90-28.95)	0.07(0.04-0.11)	18.85(11.08-29.80)	0.08(0.04-0.12)	0.07(-0.08-0.23)
Virgin Islands, U.S.	0.03(0.02-0.04)	0.08(0.05-0.13)	0.01(0.00-0.02)	0.07(0.03-0.15)	0.75(0.23-1.27)
Yemen	0.19(0.04-0.96)	0.00(0.00-0.01)	0.93(0.34-3.18)	0.01(0.00-0.02)	4.20(3.59-4.81)
Zambia	7.57(4.34-12.76)	0.20(0.12-0.34)	16.61(8.77-29.03)	0.20(0.11-0.35)	0.42(0.08-0.76)

Zimbabwe	2.25(1.10-3.69)	0.05(0.02-0.08)	5.10(2.83-8.03)	0.08(0.04-0.13)	2.14(1.68-2.60)

Abbreviations: EAPC, estimated annual percentage change; UI, uncertainty interval.

[a] EAPC is expressed as 95% confidence interval.

Table S5. DALYs of neuroblastoma in children, by countries and territories, 1990-2021.

	Rate per 100 000 (95%UI)				
	1990		2021		1990-2021
location	Number of DALYs	DALYs rate	Number of DALYs	DALYs rate	EAPC [a]
Afghanistan	9.90(1.57-52.34)	0.23(0.04-1.21)	73.94(26.57-264.26)	0.52(0.19-1.86)	3.22(2.79-3.65)
Albania	44.09(28.04-62.43)	3.95(2.51-5.59)	21.53(8.95-37.91)	4.85(2.02-8.54)	0.56(0.27-0.85)
Algeria	1140.99(664.13-1870.72)	10.64(6.19-17.44)	1027.13(637.75-1675.13)	7.72(4.79-12.59)	-0.56(-0.80, -0.32)
American Samoa	0.02(0.01-0.03)	0.08(0.05-0.14)	0.01(0.00-0.02)	0.07(0.03-0.13)	-0.85(-1.69-0.00)
Andorra	2.06(1.21-3.44)	21.71(12.69-36.25)	0.82(0.48-1.27)	8.05(4.75-12.50)	-2.62(-3.00, -2.24)
Angola	136.17(40.44-356.20)	2.89(0.86-7.55)	399.96(228.35-707.67)	2.62(1.50-4.64)	-0.01(-0.19-0.18)
Antigua and Barbuda	1.61(0.86-2.66)	8.84(4.70-14.60)	2.01(1.29-3.09)	11.87(7.62-18.27)	1.19(0.77-1.61)
Argentina	1224.21(933.94-1633.22)	12.08(9.21-16.11)	1258.59(880.59-1746.00)	12.36(8.65-17.15)	0.57(0.17-0.98)
Armenia	14.47(6.81-28.49)	1.39(0.65-2.73)	17.43(8.77-30.03)	2.94(1.48-5.07)	4.33(3.62-5.06)

Australia	558.16(469.87-662.30)	14.74(12.41-17.49)	591.21(429.35-806.82)	12.45(9.04-16.99)	-0.50(-0.76, -0.25)
Austria	223.70(193.21-264.18)	16.59(14.33-19.59)	113.70(78.40-157.72)	8.77(6.04-12.16)	-1.66(-1.84, -1.48)
Azerbaijan	49.80(26.17-85.27)	2.05(1.08-3.51)	40.19(22.27-67.33)	1.70(0.94-2.85)	0.47(-0.02-0.96)
Bahrain	4.26(2.36-7.75)	2.61(1.44-4.75)	16.38(9.29-26.05)	5.52(3.13-8.78)	4.44(3.60-5.29)
Bangladesh	2954.94(1592.47-4812.16)	6.04(3.26-9.84)	2937.31(1768.94-4566.02)	6.42(3.87-9.98)	0.22(-0.05-0.48)
Barbados	18.68(13.41-25.23)	29.96(21.51-40.46)	12.37(8.10-19.01)	26.28(17.20-40.36)	-0.14(-0.34-0.06)
Belarus	258.82(174.61-370.46)	10.77(7.26-15.41)	93.55(60.88-144.65)	5.93(3.86-9.17)	-1.45(-1.89, -1.01)
Belgium	299.61(237.41-376.19)	16.59(13.14-20.83)	220.35(153.24-303.20)	11.52(8.01-15.86)	-1.47(-1.66, -1.29)
Belize	14.50(10.49-19.93)	17.71(12.82-24.35)	20.35(14.44-28.72)	16.53(11.73-23.33)	0.08(-0.82-0.98)
Benin	40.61(18.80-73.71)	1.68(0.78-3.04)	17.57(8.14-42.03)	0.29(0.13-0.69)	-6.46(-7.12, -5.80)
Bermuda	0.51(0.33-0.81)	4.25(2.77-6.77)	0.59(0.40-0.86)	7.03(4.74-10.22)	2.37(1.40-3.35)
Bhutan	11.61(4.25-25.29)	4.43(1.62-9.64)	12.57(5.47-23.30)	6.71(2.92-12.45)	1.61(1.34-1.87)
Bolivia	322.78(184.71-529.55)	12.02(6.88-19.72)	348.60(217.06-527.51)	10.00(6.23-15.13)	-0.59(-0.71, -0.47)

Bosnia and Herzegovina	39.22(22.99-65.63)	3.58(2.10-5.99)	18.82(11.62-28.44)	3.84(2.37-5.80)	1.27(0.83-1.70)
Botswana	16.11(9.52-27.08)	2.73(1.61-4.59)	44.54(22.65-89.87)	6.38(3.24-12.87)	3.49(3.07-3.90)
Brazil	8456.74(7249.76-9689.89)	16.28(13.96-18.65)	7273.83(5549.49-9006.18)	15.10(11.52-18.69)	0.21(-0.29-0.72)
Brunei	12.71(7.98-20.72)	14.04(8.81-22.88)	10.86(7.18-16.02)	11.48(7.59-16.94)	-0.16(-0.49-0.16)
Bulgaria	110.18(60.73-179.70)	6.35(3.50-10.35)	53.26(27.86-91.81)	5.46(2.85-9.41)	-1.05(-1.80, -0.30)
Burkina Faso	47.81(19.88-93.43)	1.01(0.42-1.98)	20.46(8.20-52.10)	0.20(0.08-0.50)	-5.88(-6.57, -5.19)
Burundi	246.03(141.06-445.16)	9.39(5.38-16.98)	306.94(95.53-755.90)	5.24(1.63-12.91)	-0.74(-1.26, -0.23)
Cambodia	114.41(56.71-189.12)	2.45(1.22-4.06)	138.92(79.41-235.18)	2.72(1.55-4.60)	0.37(0.15-0.60)
Cameroon	98.20(43.68-179.74)	2.01(0.89-3.68)	46.57(23.01-132.13)	0.35(0.17-0.98)	-6.18(-6.79, -5.56)
Canada	1077.96(869.06-1333.82)	18.74(15.11-23.19)	750.85(518.87-1154.17)	12.17(8.41-18.70)	-1.23(-1.50, -0.95)
Cape Verde	0.01(0.00-0.04)	0.01(0.00-0.02)	0.02(0.01-0.07)	0.02(0.01-0.05)	3.36(3.09-3.63)
Central African Republic	24.00(9.62-48.28)	1.96(0.79-3.95)	36.02(16.88-69.40)	1.58(0.74-3.04)	-0.46(-0.65, -0.28)
Chad	23.67(10.66-44.79)	0.81(0.36-1.53)	15.02(5.94-43.91)	0.17(0.07-0.49)	-5.83(-6.51, -5.15)

Chile	145.79(117.61-180.47)	3.67(2.96-4.54)	273.39(203.48-366.81)	7.49(5.57-10.04)	2.49(1.35-3.65)
China	16801.33(12134.28-22440.18)	5.28(3.81-7.05)	14240.14(10185.93-18731.63)	5.48(3.92-7.21)	1.06(0.61-1.51)
Colombia	1102.44(839.65-1399.30)	9.45(7.20-12.00)	1029.70(694.15-1444.61)	9.70(6.54-13.61)	0.71(-0.00-1.43)
Comoros	25.10(12.38-45.03)	11.80(5.82-21.17)	38.59(18.35-71.72)	16.07(7.64-29.86)	0.86(0.59-1.12)
Congo	41.49(17.52-79.45)	3.94(1.66-7.55)	62.08(34.39-105.57)	3.22(1.78-5.47)	-0.47(-0.68, -0.25)
Cook Islands	0.01(0.00-0.03)	0.22(0.07-0.52)	0.02(0.00-0.06)	0.52(0.09-1.56)	0.10(-0.86-1.06)
Costa Rica	138.19(103.99-179.53)	12.29(9.25-15.97)	71.96(51.29-100.79)	7.07(5.04-9.91)	-1.43(-1.66, -1.19)
Cote d'Ivoire	179.13(40.29-338.61)	3.14(0.71-5.94)	387.64(97.21-822.63)	3.35(0.84-7.11)	0.05(-0.32-0.42)
Croatia	120.61(93.24-152.20)	12.22(9.45-15.42)	77.45(52.88-108.42)	12.97(8.86-18.16)	0.17(-0.13-0.46)
Cuba	330.24(246.85-443.49)	13.19(9.86-17.71)	204.09(144.29-281.69)	11.49(8.12-15.85)	0.74(0.19-1.30)
Cyprus	21.68(13.87-34.88)	10.95(7.01-17.62)	22.74(14.73-35.69)	10.40(6.74-16.32)	0.86(0.35-1.37)
Czech Republic	202.88(155.80-272.18)	9.21(7.07-12.35)	124.62(79.34-190.26)	7.26(4.62-11.09)	-1.08(-1.51, -0.64)
Democratic Republic of the Congo	407.55(162.37-792.59)	2.30(0.92-4.48)	458.75(263.79-757.89)	1.21(0.69-1.99)	-1.57(-2.16, -0.98)

Denmark	60.57(44.03-80.06)	6.86(4.98-9.06)	129.07(87.29-188.38)	13.53(9.15-19.74)	1.33(0.83-1.82)
Djibouti	29.01(13.07-50.84)	16.66(7.51-29.20)	61.30(25.38-116.07)	14.84(6.14-28.09)	-0.00(-0.32-0.31)
Dominica	2.06(1.21-3.28)	8.30(4.87-13.20)	3.39(1.63-6.35)	24.74(11.88-46.40)	3.51(3.11-3.91)
Dominican Republic	191.02(111.46-433.99)	7.09(4.14-16.10)	522.99(317.22-818.24)	17.80(10.80-27.85)	1.63(0.82-2.45)
Ecuador	225.10(166.16-303.85)	5.82(4.30-7.86)	329.56(224.06-494.89)	6.50(4.42-9.76)	0.86(0.26-1.46)
Egypt	844.42(363.66-1962.02)	3.81(1.64-8.85)	1168.24(698.77-2319.64)	3.17(1.90-6.29)	-0.09(-0.81-0.64)
El Salvador	100.02(67.19-142.42)	4.63(3.11-6.60)	60.00(40.42-85.97)	3.30(2.22-4.73)	-1.28(-1.49, -1.06)
Equatorial Guinea	4.26(1.83-8.53)	2.16(0.93-4.33)	23.99(10.99-45.55)	4.10(1.88-7.79)	2.12(1.73-2.51)
Eritrea	124.17(69.15-205.75)	7.80(4.34-12.92)	327.92(145.59-629.50)	12.99(5.77-24.93)	1.54(1.24-1.85)
Estonia	37.62(25.24-57.69)	10.78(7.23-16.53)	12.00(7.27-20.03)	5.55(3.36-9.27)	-1.48(-1.79, -1.17)
Ethiopia	1596.84(647.29-5312.57)	6.55(2.66-21.81)	3801.53(1602.09-10697.57)	8.57(3.61-24.12)	0.93(0.34-1.52)
Federated States of Micronesia	0.10(0.04-0.21)	0.22(0.08-0.46)	0.06(0.02-0.13)	0.19(0.07-0.41)	-0.65(-0.83, -0.47)
Fiji	3.99(1.56-9.33)	1.42(0.55-3.31)	4.02(1.41-9.86)	1.48(0.52-3.62)	-1.91(-3.18, -0.62)

Finland	48.55(38.22-61.43)	5.03(3.96-6.37)	142.45(103.15-196.67)	16.82(12.18-23.22)	3.56(2.74-4.38)
France	1799.50(1543.28-2045.04)	15.36(13.17-17.46)	1210.84(891.28-1671.68)	10.43(7.68-14.40)	-1.22(-1.44, -1.00)
Gabon	14.52(7.68-28.61)	3.56(1.88-7.02)	27.14(15.38-44.79)	4.25(2.41-7.01)	0.93(0.71-1.16)
Georgia	4.31(2.50-7.48)	0.32(0.18-0.55)	26.39(15.35-41.78)	3.59(2.09-5.68)	12.60(11.05-14.18)
Germany	1686.57(1340.54-2120.20)	13.03(10.35-16.38)	1759.63(1292.19-2378.82)	14.71(10.80-19.88)	0.20(-0.12-0.53)
Ghana	6.64(3.54-12.45)	0.10(0.05-0.19)	5.91(1.98-18.71)	0.05(0.02-0.15)	-3.99(-5.19, -2.78)
Global	145057.36(120924.76-173294.30)	8.34(6.95-9.96)	174186.30(127104.64-223265.92)	8.66(6.32-11.10)	0.45(0.32-0.57)
Greece	125.12(108.61-142.79)	6.18(5.37-7.06)	76.12(59.81-94.53)	5.46(4.29-6.78)	0.28(-0.06-0.61)
Greenland	1.98(1.27-2.91)	13.95(8.94-20.45)	0.26(0.13-0.66)	2.23(1.10-5.60)	-5.84(-6.20, -5.49)
Grenada	2.38(1.55-3.75)	7.13(4.64-11.23)	2.12(1.37-3.26)	9.71(6.28-14.91)	1.65(1.21-2.08)
Guam	0.07(0.04-0.11)	0.17(0.10-0.27)	0.08(0.04-0.13)	0.22(0.12-0.36)	2.88(1.52-4.26)
Guatemala	165.21(101.38-274.97)	4.07(2.50-6.77)	132.74(91.32-188.30)	2.69(1.85-3.82)	-1.48(-1.74, -1.21)
Guinea	259.35(67.55-456.35)	9.42(2.45-16.58)	466.02(112.09-1012.22)	7.71(1.85-16.74)	-0.40(-0.63, -0.16)

Guinea-Bissau	7.60(3.71-13.27)	1.58(0.77-2.75)	1.68(0.70-4.19)	0.19(0.08-0.47)	-7.39(-7.79, -6.98)
Guyana	2.79(1.86-4.01)	0.95(0.63-1.37)	34.73(21.78-53.75)	16.28(10.21-25.19)	7.96(5.52-10.46)
Haiti	342.94(102.58-699.30)	12.64(3.78-25.78)	690.06(284.45-1294.38)	15.85(6.53-29.74)	1.28(1.11-1.45)
Honduras	134.03(83.85-207.90)	6.07(3.80-9.41)	166.85(96.01-275.97)	5.09(2.93-8.42)	-0.67(-0.84, -0.49)
Hungary	218.68(172.98-269.93)	10.26(8.12-12.67)	115.24(78.34-164.81)	8.30(5.64-11.87)	-0.59(-1.26-0.09)
Iceland	7.44(5.77-9.39)	11.73(9.10-14.81)	8.36(5.27-13.40)	12.38(7.80-19.85)	1.18(0.49-1.88)
India	20271.84(12412.82-28015.45)	6.21(3.80-8.58)	23408.65(15694.05-32008.67)	6.39(4.28-8.74)	0.05(-0.30-0.40)
Indonesia	2482.59(1383.45-4328.24)	3.67(2.04-6.39)	3395.93(2318.89-5191.10)	5.05(3.45-7.71)	0.91(0.78-1.04)
Iran	145.51(81.12-260.14)	0.57(0.32-1.02)	229.03(96.63-366.36)	1.13(0.48-1.82)	4.84(3.89-5.80)
Iraq	370.22(227.11-583.87)	4.49(2.76-7.09)	929.92(567.68-1513.15)	6.91(4.22-11.24)	1.88(1.64-2.11)
Ireland	150.15(123.32-186.54)	15.28(12.55-18.99)	79.30(55.33-110.95)	7.95(5.55-11.13)	-1.34(-1.67, -1.01)
Israel	287.85(209.33-373.24)	18.78(13.65-24.35)	288.39(203.18-395.39)	10.97(7.73-15.05)	-1.49(-1.86, -1.12)
Italy	2470.92(2232.78-2704.29)	26.77(24.19-29.30)	1615.79(1220.64-2040.69)	21.26(16.06-26.85)	-0.78(-1.07, -0.50)

Jamaica	116.18(87.77-155.62)	13.91(10.51-18.63)	141.09(85.52-220.67)	24.16(14.65-37.79)	1.95(1.17-2.75)
Japan	4587.29(4370.00-4795.51)	19.87(18.93-20.77)	2642.30(2334.09-2945.57)	17.11(15.11-19.07)	-0.74(-1.11, -0.37)
Jordan	150.30(98.17-231.58)	9.20(6.01-14.18)	374.53(243.79-573.38)	10.31(6.71-15.78)	0.48(0.21-0.74)
Kazakhstan	37.91(22.80-58.97)	0.73(0.44-1.13)	56.23(32.76-90.05)	1.04(0.60-1.66)	1.83(1.57-2.09)
Kenya	425.65(254.89-760.18)	3.81(2.28-6.81)	864.11(474.15-1395.47)	4.63(2.54-7.48)	1.17(0.88-1.46)
Kiribati	0.03(0.02-0.06)	0.12(0.06-0.22)	0.06(0.02-0.13)	0.15(0.06-0.30)	0.42(0.21-0.63)
Kuwait	43.59(31.80-59.29)	7.86(5.74-10.70)	72.34(47.26-104.86)	8.56(5.59-12.40)	3.98(1.85-6.15)
Kyrgyzstan	112.87(63.00-190.61)	6.73(3.76-11.36)	28.10(18.86-41.06)	1.24(0.83-1.81)	1.79(-1.30-4.98)
Laos	39.17(14.59-78.86)	2.13(0.79-4.28)	71.47(39.69-122.15)	3.11(1.73-5.32)	1.51(1.37-1.65)
Latvia	50.46(33.49-79.65)	8.87(5.89-14.00)	7.22(4.09-12.09)	2.43(1.38-4.07)	-2.15(-2.94, -1.35)
Lebanon	58.01(36.11-88.24)	5.55(3.45-8.44)	78.08(38.81-137.83)	6.11(3.04-10.78)	0.89(0.64-1.14)
Lesotho	11.51(6.69-19.73)	1.69(0.98-2.89)	15.80(8.89-26.86)	2.51(1.41-4.26)	1.74(1.29-2.19)
Liberia	19.99(10.18-33.44)	1.77(0.90-2.96)	3.36(1.28-8.13)	0.15(0.06-0.37)	-8.00(-9.00, -6.99)

Libya	188.81(107.11-341.78)	10.43(5.91-18.87)	239.39(129.03-407.90)	16.05(8.65-27.35)	1.81(1.36-2.27)
Lithuania	61.08(39.49-91.97)	7.35(4.75-11.07)	19.60(12.33-28.54)	4.81(3.02-7.00)	-0.65(-1.09, -0.20)
Luxembourg	11.25(9.38-13.32)	17.03(14.20-20.17)	8.21(6.16-10.68)	8.11(6.09-10.55)	-3.11(-3.50, -2.71)
Macedonia	46.67(32.47-66.68)	8.86(6.16-12.66)	14.68(8.55-23.04)	4.48(2.61-7.03)	-0.85(-1.31, -0.38)
Madagascar	674.82(418.50-1054.29)	12.37(7.67-19.32)	1096.10(523.87-1949.11)	9.34(4.46-16.61)	-0.60(-0.97, -0.23)
Malawi	2024.39(1310.35-3042.82)	44.50(28.80-66.88)	3033.67(1140.63-6370.25)	37.34(14.04-78.41)	-0.28(-0.51, -0.05)
Malaysia	414.19(231.64-628.32)	6.30(3.52-9.56)	573.60(379.75-805.11)	7.53(4.99-10.57)	0.80(0.33-1.28)
Maldives	15.56(6.39-38.36)	14.81(6.08-36.52)	22.41(14.25-34.09)	22.37(14.23-34.03)	2.04(1.67-2.42)
Mali	154.71(32.19-290.55)	3.75(0.78-7.04)	356.86(79.75-809.20)	3.08(0.69-6.99)	-0.67(-0.88, -0.46)
Malta	26.42(19.18-35.52)	30.18(21.92-40.59)	20.63(13.08-30.71)	32.23(20.43-47.98)	0.17(-0.26-0.61)
Marshall Islands	0.03(0.01-0.07)	0.13(0.05-0.30)	0.04(0.02-0.08)	0.22(0.09-0.46)	1.51(1.10-1.92)
Mauritania	16.49(7.84-30.32)	1.78(0.85-3.28)	5.54(2.28-16.46)	0.30(0.12-0.89)	-7.00(-7.57, -6.42)
Mauritius	9.88(8.15-11.97)	2.99(2.47-3.63)	13.37(10.06-16.59)	6.44(4.85-8.00)	2.50(1.53-3.48)

Mexico	3646.87(3277.16-4115.37)	10.91(9.81-12.32)	2518.89(1926.22-3369.57)	7.86(6.01-10.51)	-0.96(-1.69, -0.23)
Moldova	49.60(35.90-74.66)	4.01(2.90-6.04)	23.71(16.50-32.14)	4.54(3.16-6.15)	1.63(1.16-2.09)
Mongolia	18.05(8.14-36.02)	2.01(0.90-4.00)	22.01(12.35-37.78)	2.03(1.14-3.48)	0.79(0.32-1.27)
Montenegro	13.82(8.90-21.01)	8.55(5.51-13.00)	4.11(2.55-6.64)	3.69(2.29-5.96)	-1.44(-1.85, -1.04)
Morocco	1202.52(733.85-1800.77)	12.29(7.50-18.40)	1202.02(671.94-2113.39)	12.28(6.86-21.58)	0.53(0.28-0.79)
Mozambique	712.60(401.93-1346.28)	11.49(6.48-21.70)	1437.33(528.86-3272.23)	10.08(3.71-22.94)	-0.22(-0.50-0.06)
Myanmar	478.74(193.88-907.49)	3.24(1.31-6.14)	753.67(458.39-1186.97)	4.83(2.94-7.60)	1.48(1.39-1.57)
Namibia	44.66(21.60-69.71)	7.43(3.60-11.60)	116.64(59.55-192.92)	14.13(7.21-23.37)	2.67(2.29-3.04)
Nepal	367.64(208.90-664.48)	4.36(2.48-7.89)	381.67(179.11-671.83)	4.14(1.94-7.28)	0.01(-0.13-0.15)
Netherlands	614.04(501.95-763.06)	22.53(18.42-28.00)	327.96(230.46-452.73)	12.23(8.59-16.88)	-2.48(-2.84, -2.11)
New Zealand	123.47(99.35-155.57)	15.43(12.42-19.44)	81.79(63.68-104.38)	8.33(6.49-10.63)	-1.18(-1.84, -0.51)
Nicaragua	318.51(206.27-482.76)	17.49(11.33-26.51)	157.94(101.94-239.39)	7.98(5.15-12.09)	-2.10(-2.41, -1.78)
Niger	42.20(18.65-88.39)	1.04(0.46-2.18)	11.50(3.92-28.53)	0.09(0.03-0.22)	-8.78(-9.52, -8.03)

Nigeria	5711.81(1565.30-9053.34)	14.60(4.00-23.14)	21873.52(5035.87-38816.44)	21.53(4.96-38.21)	1.68(1.29-2.06)
North Korea	324.25(191.78-529.36)	5.45(3.22-8.90)	133.50(59.83-265.44)	2.80(1.25-5.56)	-1.73(-2.00, -1.45)
Northern Mariana Islands	0.00(0.00-0.01)	0.02(0.01-0.06)	0.00(0.00-0.01)	0.04(0.01-0.08)	3.05(1.95-4.17)
Norway	83.27(76.10-90.02)	10.43(9.53-11.28)	40.80(33.30-50.22)	4.42(3.60-5.44)	-3.47(-3.92, -3.02)
Oman	36.89(10.39-75.93)	4.39(1.24-9.03)	53.09(32.04-77.11)	4.34(2.62-6.30)	0.54(-0.12-1.21)
Pakistan	7467.57(4692.13-11816.85)	15.17(9.53-24.00)	23878.68(14590.73-37924.28)	27.95(17.08-44.39)	2.97(2.57-3.37)
Palestine	65.42(36.77-120.25)	6.76(3.80-12.42)	171.50(104.64-259.25)	9.18(5.60-13.88)	2.21(1.65-2.78)
Panama	102.24(79.25-129.44)	12.26(9.50-15.52)	124.85(93.43-167.15)	10.82(8.10-14.49)	-0.76(-1.01, -0.52)
Papua New Guinea	2.19(0.73-4.42)	0.13(0.04-0.26)	6.44(2.94-12.49)	0.16(0.08-0.32)	0.59(0.31-0.87)
Paraguay	156.88(94.65-239.99)	9.40(5.67-14.37)	223.44(131.82-370.35)	11.13(6.57-18.45)	0.96(0.67-1.26)
Peru	991.44(628.83-1399.46)	11.94(7.58-16.86)	507.17(321.06-777.12)	5.32(3.37-8.15)	-2.08(-2.27, -1.89)
Philippines	1809.61(1219.44-2654.50)	7.18(4.84-10.53)	2555.11(1859.36-3274.08)	7.52(5.47-9.63)	0.66(0.47-0.85)
Poland	779.33(710.23-854.25)	8.14(7.42-8.92)	362.38(293.16-440.86)	6.16(4.98-7.49)	-0.79(-1.80-0.24)

Portugal	577.19(467.91-695.09)	27.28(22.11-32.85)	175.23(128.07-246.60)	12.86(9.40-18.10)	-2.84(-3.10, -2.58)
Principality of Monaco	0.01(0.01-0.01)	0.24(0.14-0.40)	0.02(0.01-0.03)	0.34(0.18-0.60)	-0.96(-1.80, -0.11)
Puerto Rico	78.65(59.37-105.20)	7.90(5.96-10.56)	25.19(18.06-35.75)	5.67(4.06-8.04)	-0.17(-0.77-0.43)
Qatar	1.23(0.79-2.03)	0.98(0.63-1.63)	6.91(3.63-11.68)	1.40(0.74-2.37)	2.92(2.02-3.83)
Republic of Nauru	0.02(0.01-0.04)	0.37(0.15-0.89)	0.02(0.01-0.04)	0.43(0.17-0.89)	0.24(0.09-0.38)
Republic of Niue	0.00(0.00-0.01)	0.45(0.20-0.88)	0.01(0.00-0.02)	2.24(1.09-4.02)	2.19(1.09-3.29)
Republic of Palau	0.00(0.00-0.00)	0.01(0.00-0.02)	0.00(0.00-0.00)	0.01(0.00-0.01)	-1.09(-1.34, -0.85)
Republic of San Marino	0.19(0.12-0.30)	4.76(3.04-7.28)	0.10(0.04-0.18)	2.18(1.02-4.21)	-2.33(-2.60, -2.05)
Romania	784.13(451.62-1337.99)	14.08(8.11-24.03)	244.37(146.65-396.14)	8.12(4.87-13.16)	-1.65(-2.04, -1.26)
Russian Federation	2977.05(2318.08-3885.58)	8.58(6.68-11.20)	1163.15(913.40-1361.62)	4.46(3.50-5.22)	-2.39(-2.65, -2.14)
Rwanda	467.28(276.27-767.35)	13.77(8.14-22.62)	581.63(249.87-1069.33)	11.70(5.03-21.51)	-0.20(-0.51-0.10)
Saint Kitts and Nevis	1.06(0.69-1.66)	7.54(4.88-11.77)	1.63(0.95-2.63)	16.52(9.62-26.72)	2.54(2.28-2.81)
Saint Lucia	4.83(3.29-7.08)	9.37(6.38-13.73)	3.86(2.30-6.15)	13.01(7.74-20.73)	1.26(1.00-1.52)

Saint Vincent and the Grenadines	0.23(0.17-0.31)	0.56(0.41-0.75)	3.09(2.12-4.53)	12.38(8.49-18.15)	6.88(3.08-10.82)
Samoa	2.56(1.21-4.90)	3.59(1.69-6.88)	3.35(1.10-7.39)	4.19(1.37-9.24)	0.50(0.36-0.64)
Sao Tome and Principe	1.16(0.55-2.16)	2.04(0.96-3.81)	0.18(0.05-0.43)	0.23(0.06-0.56)	-7.37(-8.41, -6.31)
Saudi Arabia	445.06(83.08-785.05)	6.79(1.27-11.98)	220.63(90.59-421.75)	2.92(1.20-5.57)	-2.31(-3.60, -1.01)
Senegal	68.05(29.31-124.31)	1.86(0.80-3.40)	15.53(4.91-35.25)	0.24(0.08-0.55)	-7.22(-7.84, -6.59)
Serbia	415.27(232.43-680.07)	19.15(10.72-31.36)	84.52(45.70-155.88)	6.37(3.44-11.74)	-4.12(-4.62, -3.63)
Seychelles	0.00(0.00-0.00)	0.00(0.00-0.00)	0.00(0.00-0.00)	0.00(0.00-0.00)	1.40(1.00-1.80)
Sierra Leone	33.06(15.26-58.20)	1.82(0.84-3.21)	8.40(3.88-21.92)	0.23(0.11-0.61)	-7.21(-8.03, -6.38)
Singapore	124.41(100.82-149.74)	19.16(15.53-23.06)	108.82(78.80-147.81)	13.40(9.70-18.20)	-0.91(-1.36, -0.46)
Slovakia	95.14(59.10-148.00)	7.18(4.46-11.16)	68.31(37.22-123.31)	7.98(4.35-14.40)	0.63(0.33-0.94)
Slovenia	26.71(21.13-33.58)	6.46(5.11-8.12)	8.52(5.62-12.62)	2.73(1.80-4.04)	-2.36(-3.04, -1.67)
Solomon Islands	0.14(0.04-0.35)	0.09(0.03-0.22)	0.32(0.09-0.73)	0.12(0.04-0.28)	0.79(0.31-1.28)
Somalia	133.72(62.39-333.74)	3.43(1.60-8.57)	192.62(65.02-571.72)	1.86(0.63-5.53)	-1.41(-1.74, -1.07)

South Africa	732.02(474.03-1060.42)	5.38(3.48-7.79)	851.59(622.99-1228.82)	5.60(4.10-8.08)	0.56(0.11-1.01)
South Korea	1914.57(1247.89-2806.53)	16.84(10.97-24.68)	557.80(350.32-824.82)	9.18(5.77-13.58)	-2.63(-3.07, -2.19)
South Sudan	246.52(110.59-506.43)	9.39(4.21-19.30)	373.23(192.57-679.21)	8.69(4.48-15.81)	0.39(-0.03-0.80)
Spain	1496.65(1298.21-1717.25)	19.10(16.57-21.91)	922.14(719.40-1174.01)	14.23(11.10-18.12)	-0.83(-1.03, -0.63)
Sri Lanka	332.49(227.48-484.12)	6.01(4.11-8.75)	286.71(169.01-471.02)	5.62(3.31-9.23)	0.05(-0.51-0.61)
Sudan	51.43(9.91-232.54)	0.58(0.11-2.62)	179.94(73.46-584.51)	1.08(0.44-3.52)	3.09(2.68-3.51)
Suriname	6.98(4.24-10.78)	5.36(3.26-8.28)	11.07(6.48-17.24)	7.73(4.52-12.03)	1.67(1.33-2.01)
Swaziland	12.73(7.39-21.99)	3.30(1.92-5.70)	18.31(10.62-33.08)	4.44(2.57-8.02)	1.18(0.70-1.66)
Sweden	260.44(206.03-328.17)	16.86(13.34-21.25)	192.55(134.73-270.59)	10.58(7.40-14.86)	-1.93(-2.50, -1.35)
Switzerland	152.38(124.52-184.44)	13.19(10.77-15.96)	257.72(184.50-358.58)	19.34(13.85-26.91)	0.41(-0.12-0.93)
Syria	96.38(50.94-186.21)	1.63(0.86-3.14)	54.52(31.36-82.83)	1.49(0.86-2.26)	0.39(-0.11-0.89)
Taiwan (Province of China)	249.95(214.89-288.00)	4.54(3.90-5.23)	160.33(126.03-211.15)	5.44(4.28-7.17)	0.22(-0.29-0.73)
Tajikistan	1.97(1.07-3.39)	0.08(0.05-0.15)	2.95(1.70-4.80)	0.08(0.05-0.13)	0.08(-0.50-0.66)

Tanzania	1735.22(1065.78-2898.27)	14.37(8.83-24.00)	4303.00(1980.35-7660.11)	17.63(8.12-31.39)	1.29(0.98-1.59)
Thailand	1199.79(795.59-1683.64)	7.12(4.72-9.99)	772.61(553.49-1062.91)	7.91(5.67-10.88)	-0.16(-0.49-0.17)
The Bahamas	10.48(7.17-14.90)	12.99(8.89-18.47)	10.44(6.82-16.37)	12.86(8.40-20.16)	0.01(-0.33-0.36)
The Gambia	26.20(6.95-48.66)	5.68(1.51-10.55)	52.35(16.07-111.95)	5.27(1.62-11.27)	-0.64(-0.98, -0.30)
Timor-Leste	7.87(3.22-17.19)	2.37(0.97-5.17)	12.73(7.44-20.83)	2.45(1.43-4.00)	-0.28(-0.61-0.05)
Togo	27.67(11.97-53.81)	1.57(0.68-3.05)	6.97(2.86-18.13)	0.21(0.09-0.55)	-7.41(-8.14, -6.68)
Tokelau	0.00(0.00-0.00)	0.23(0.08-0.52)	0.01(0.00-0.03)	3.01(0.77-8.02)	2.79(0.55-5.08)
Tonga	0.06(0.02-0.13)	0.15(0.06-0.31)	0.08(0.03-0.18)	0.21(0.08-0.46)	0.66(0.25-1.06)
Trinidad and Tobago	111.64(87.82-140.35)	27.48(21.61-34.54)	91.33(63.93-126.26)	33.53(23.47-46.35)	1.53(1.14-1.92)
Tunisia	486.55(305.97-766.96)	15.67(9.85-24.70)	332.10(192.59-580.51)	12.01(6.96-20.99)	-0.39(-0.60, -0.18)
Turkey	3349.25(1941.47-5392.64)	16.35(9.48-26.32)	3417.85(2141.22-5084.91)	18.45(11.56-27.46)	0.92(0.58-1.26)
Turkmenistan	25.07(13.47-47.69)	1.67(0.90-3.18)	34.29(20.51-55.98)	2.25(1.35-3.67)	1.48(1.12-1.83)
Tuvalu	0.01(0.00-0.02)	0.23(0.09-0.44)	0.01(0.00-0.02)	0.21(0.09-0.44)	-0.39(-0.62, -0.16)

Uganda	938.18(490.12-1620.15)	11.14(5.82-19.24)	3230.29(1408.61-6314.28)	16.29(7.10-31.83)	1.55(1.26-1.83)
Ukraine	1043.20(699.63-1580.29)	9.17(6.15-13.89)	555.16(338.58-862.20)	8.75(5.34-13.59)	0.75(0.23-1.28)
United Arab Emirates	23.40(12.82-43.14)	3.97(2.18-7.32)	48.12(30.23-72.00)	3.59(2.26-5.38)	0.82(0.24-1.41)
United Kingdom	3340.12(3189.79-3508.49)	30.59(29.21-32.13)	1530.32(1333.53-1702.63)	12.99(11.32-14.45)	-2.12(-2.64, -1.59)
United States	11211.64(10328.74-12068.24)	20.05(18.47-21.58)	7174.10(6125.32-8219.63)	12.07(10.31-13.83)	-1.33(-1.51, -1.14)
Uruguay	101.97(77.50-137.34)	12.46(9.47-16.78)	74.12(51.45-105.00)	11.24(7.80-15.92)	-0.48(-0.81, -0.15)
Uzbekistan	184.24(106.37-320.27)	2.15(1.24-3.74)	399.60(236.78-627.08)	3.96(2.35-6.21)	2.78(2.45-3.11)
Vanuatu	0.06(0.02-0.14)	0.09(0.03-0.21)	0.14(0.05-0.32)	0.12(0.04-0.27)	1.04(0.56-1.52)
Venezuela	871.45(726.07-1057.52)	12.28(10.24-14.91)	624.66(436.89-886.73)	9.43(6.60-13.39)	-0.49(-0.89, -0.10)
Vietnam	1744.23(963.42-2593.44)	6.58(3.63-9.78)	1667.95(969.49-2642.90)	6.74(3.92-10.67)	0.06(-0.10-0.21)
Virgin Islands, U.S.	2.37(1.40-3.59)	7.41(4.39-11.22)	0.87(0.38-1.82)	6.46(2.87-13.62)	0.78(0.26-1.29)
Yemen	17.02(3.75-84.77)	0.24(0.05-1.19)	81.32(29.32-275.75)	0.59(0.21-2.00)	4.17(3.56-4.79)
Zambia	677.92(386.08-1139.09)	18.06(10.28-30.34)	1478.10(779.60-2584.82)	17.87(9.43-31.25)	0.40(0.06-0.74)

Zimbabwe	200.71(97.87-327.72)	4.17(2.03-6.80)	451.52(249.04-711.05)	7.17(3.96-11.30)	2.10(1.64-2.56)

Abbreviations: DALYs, disability adjusted life years; EAPC, estimated annual percentage change; UI, uncertainty interval.

[a] EAPC is expressed as 95% confidence interval.
